# Source identification and risk assessment of polycyclic aromatic hydrocarbons (PAHs) in air and dust samples of Lahore City

**DOI:** 10.1038/s41598-022-06437-8

**Published:** 2022-02-14

**Authors:** Rabia Aslam, Faiza Sharif, Mujtaba Baqar, Laila Shahzad

**Affiliations:** Sustainable Development Study Centre, Government College University, Lahore, Pakistan

**Keywords:** Environmental sciences, Biomarkers, Risk factors

## Abstract

During two consecutive summer and winter seasons in Lahore, the health risk of air and dust-borne polycyclic aromatic hydrocarbons (PAHs) was evaluated. Gas chromatography/mass spectrometry (GS/MS) was used to determine air and dust samples from various functional areas across the city. The mean ∑_16_PAHs were higher in air 1035.8 ± 310.7 (pg m^−3^) and dust 963.4 ± 289.0 (ng g^−1^ d.w.) during winter seasons as compared to summer seasons in air 1010.9 ± 303.3 (pg m^−3^) and dust matrices 945.2 ± 283.6 (ng g^−1^ d.w.), respectively. PAHs ring profile recognized 3 and 4 rings PAHs as most dominant in air and dust samples. Estimated results of incremental lifetime cancer risk (ILCR) highlighted high carcinogenic risk among the residents of Lahore via ingestion and dermal contact on exposure to atmospheric PAHs. The total ILCR values in air among children (summer: 9.61E − 02, winter: 2.09E − 02) and adults (summer: 1.45E − 01, winter: 3.14E − 02) and in dust, children (summer: 9.16E − 03, winter: 8.80E − 03) and adults (summer: 1.38E − 02, winter: 1.33E − 02) during the study period. The isomeric ratios in the study area revealed mixed PAH sources, including vehicular emission, petroleum, diesel and biomass combustion. As a result, it is advised that atmospheric PAHs should be monitored throughout the year and the ecologically friendly fuels be used to prevent PAHs pollution and health concerns in the city. The findings of this study are beneficial to the local regulating bodies in terms of controlling the exposure and promoting steps to reduce PAHs pollution and manage health in Lahore.

## Introduction

Polycyclic aromatic hydrocarbons (PAHs) are semi-volatile organic compounds (SVOCs) originating from various pyrogenic and petrogenic sources, including combustion and petroleum products^1, 2^. PAHs are arranged in 2–6 linear or cluster form rings^3, 4^, classified as low molecular weight (LMW) PAHs (less than four rings), and high molecular weight (HMW) PAHs (4–6 rings)^5–7^. In recent years, the PAHs have been detected in a number of media, including air, sediments, soils, and dust^8–10^. Air and dust are considered to be the major natural sinks and reservoirs of PAHs^11^. PAHs are acquiring more attention due to their carcinogenic and mutagenic properties causing adverse human health effects^12^.

United States Environmental Protection Agency (US EPA) recognized 16 polycyclic aromatic hydrocarbons as "Consent Decree" priority pollutants^5, 13^, of which 7 pollutants have been classified as potential human carcinogens: Benz(a)anthracene (BaA), Benzo(a)pyrene (BaP), Benzo(b)fluoranthene (BbF), Benzo(k)fluoranthene (BkF), Chrysene (Chry), Dibenz(ah)anthracene (DahA), and Indeno(1,2,3-cd) pyrene (IP)^14^. With the economic and industrial development of the urban environment, these pollutants' atmospheric concentrations have increased^1, 2^. A huge contribution of atmospheric PAHs includes vehicular emissions, power generation by oil and coal combustion, industrial plants, and residential heating^15^.

Absorption of contaminated foods, inhalation of polluted air and ingestion of dust are the most common routes of human exposure to PAHs^16, 17^. Among these exposure pathways, inhalation primarily refers to active and passive smoking and breathing in polluted indoor and outdoor environments^18^. Dermal exposure occurs when PAHs come into direct contact with the skin and eyes due to occupational or other environmental conditions^19^. Another major element of exposure in humans is dietary consumption of PAHs from various food categories (including fruits, vegetables, and meat)^20^. There is growing worried about the health effects of PAHs^21^. They cause severe respiratory and cardiovascular disorders, reduce lung capacity, myocardial infarction, asthma, and possibly cancer, as well as immune system failure^10, 12, 22^.

Pyrosynthesis and pyrolysis are two basic processes resulting in PAH formation in an oxygen-deficient environment from saturated hydrocarbons^23^. PAHs are present in both the gas and particulate phases of the atmosphere, where they are linked with particles^24^. Several studies have indicated that gas-phase PAHs concentrations are substantially higher than solid-phase PAHs concentrations^25, 26^. The PAHs distribution and persistence in the air are regulated by their physicochemical properties and climate conditions^27, 28^. Their toxicity depends on particulate size, molecular structure, chemical composition, and meteorology of the region^29^.

PAHs settle in dust because of dry and wet atmospheric deposition^30^ and function as natural sinks to store the organic chemicals in rural and urban environments^31^. In everyday life of residential and occupational settings, the pollutants are ingested by people through skin contact, dust absorption and inhalation^32, 33^. Much emphasis has recently been placed on the importance of polluted air and dust inhalation as a route to PAHs exposure in humans^16, 17, 34^. The size of street dust particle5 influences pAHs' structure and chemical composition. Larger dust particles often have less surface area for the deposition of PAHs. Therefore, smaller dust particles contain more PAHs than bigger ones^35^. Furthermore, due to their high hydrophobicity, PAHs with more molecular mass sink into the settled dust^36, 37^.

Pakistan, a developing South Asian nation with a population of 200 million and an annual growth rate of 2.4%, ranks seventh in the world regarding PAHs emissions^38^. Air pollution is an emerging issue in the big cities of Pakistan. The primary sources are growing urbanization, development of infrastructures, increasing industrial activities without appropriate air emission treatment or controls, transportation congestion, and vehicular exhaust^22^. Traffic pollution is one of the country's most significant man-made PAHs sources, accounting for 60% of the total PAHs released into the urban environment^39^.

Previously, only a few researches on PAHs toxicity in the environment and matrix of dust had been performed in Pakistan. Smith et al.^40^ carried out the first systematic survey in Lahore, Pakistan. Almost after 20 years, a study was conducted in Lahore, Gujranwala, and Rawalpindi showed that LMW PAHs were the most prevalent congeners, originating from the local petroleum refinery and vehicular emissions, respectively^9^. Furthermore, some epidemiological investigations on PAHs emissions in Pakistan from diverse occupational contexts like traffic wardens, brick kiln employees and workers from the petroleum industry were conducted^10, 41, 42^. However, no comprehensive research on air and dust PAHs with seasonal changes was performed in this PAHs pollution hotspot. Lahore has rapidly urbanized, with vehicle ownership and population density rising. On the other hand, the city has the initiative to monitor air quality for PAHs levels to manage air contamination^5^. To monitor the source and extent of PAHs concentrations to mitigate the environmental public health threats, a quantitative understanding of the possible sources of these pollutants in urban air and dust is needed^12, 36^. Therefore, the current study aimed to establish the distribution and levels of the profile of PAHs in air and dust samples of ten functional areas of Lahore city, assessing the seasonal variation in PAHs concentrations pattern and estimating the incremental lifetime cancer and non-cancer risks of PAHs through inhalation, ingestion and dermal routes of exposure.

## Materials and methods

### Study area

**Figure 1:**
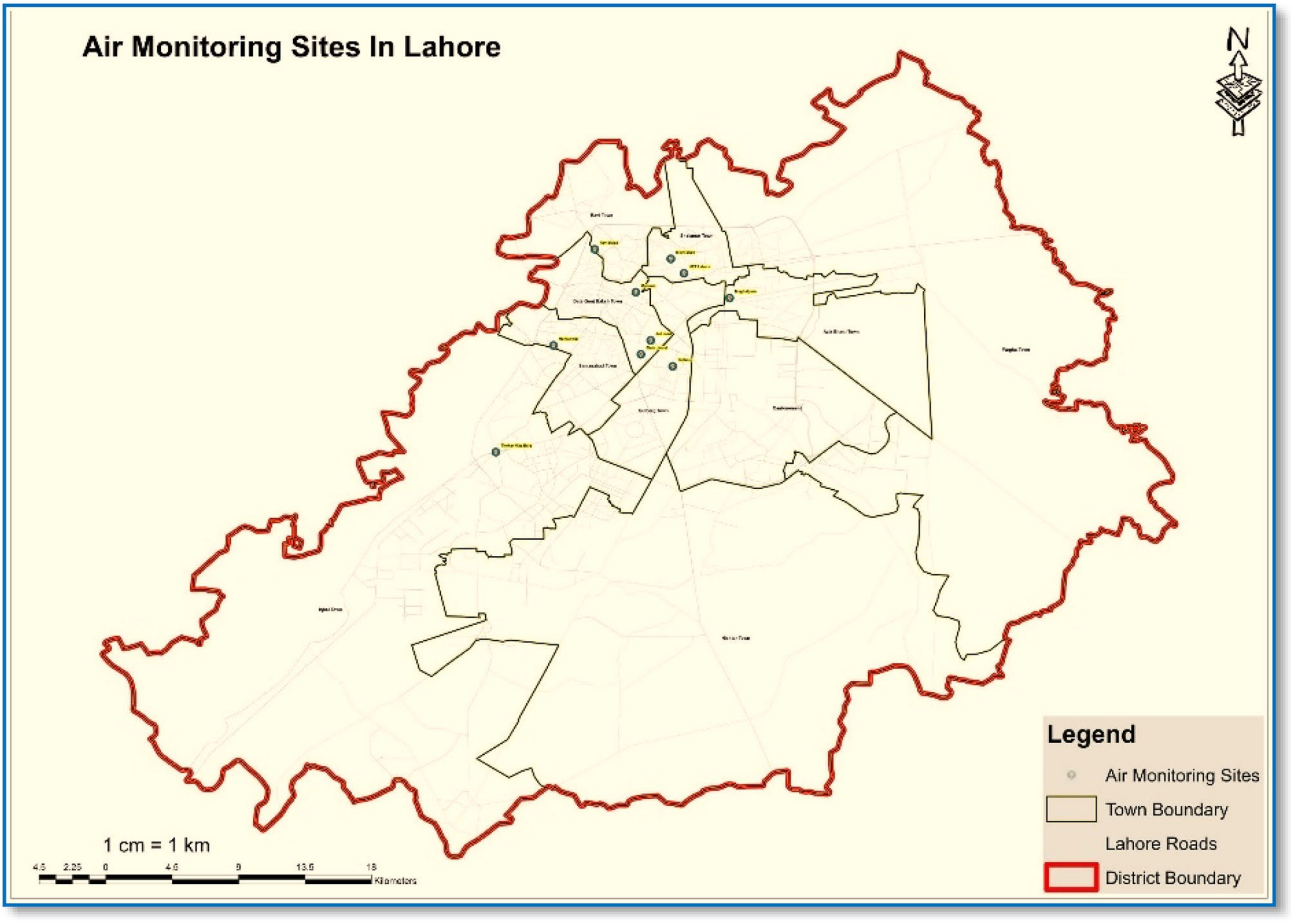
Fixed air monitoring sites, areas of respondents and roads the of study area (developed on ARC-GIS version 10.3).

Lahore is Pakistan's second most populous city^35^. According to the 2017 census, Lahore's total population is 11.13 million, with a land area of about 1772.43 km^2^ ^36^. It is situated in the Punjab province between latitudes 31° 20′ and 31° 50′ N and longitudes 74° 05′ and 74° 37′ E^43, 44^. Around 82% of the population resides in the city, and the remaining 18% lives in the rural areas around the city^45^. Lahore is situated at 63.0936 m in height above sea level. It has hot and semi-arid climate and is classified by the Köppen classification as a desert climate^46^. The average temperature is 24.1 °C, and 75.28 °F and 607–23.9 mm is annual rainfall recorded per year in Lahore^47^. The city has expanded due to a population movement and grown through a population shift for better sociocultural and economic reasons^31^. Figure 1 represents the map of the study area showing sampling sites of Lahore.

### Sample collection

#### Air sampling

For PAHs monitoring in ambient air, ten sampling sites (Shah Jamal, UET, Ravi Road, Jail Road, Thokar Niaz Baig, Misri Shah, Manawa, Gulberg, Mohlanwal and Mughalpura) were selected in Lahore city, Pakistan and total 10 passive air samplers were deployed (one at each site) for consecutive 56-days during two sampling periods, winter (October–March) and summer (April–September) in year 2017 and 2018, respectively^48^. Based on population density, high traffic congestion and intensified anthropogenic activities, ten major hotspot areas of Lahore city were identified for collecting air and dust samples. The sampling locations were chosen by dividing the city into ten most inhabited residential and commercial sectors, going through heavy vehicular and industrial pollution, affecting the public health and environment of Lahore. Another criterion for selecting these areas was that they are fixed air monitoring sites of the Punjab Environment Protection Department (EPD), from where daily concentrations of major air pollutants such as carbon monoxide (CO), nitrogen dioxide (NO_2_), sulfur dioxide (SO_2_), Ozone (O_3_), and particulate matter (PM_2.5_ and PM_10_) are monitored, considering these areas to be the most polluted in the city.

The Polyurethane Foam (PUF) discs (14 cm in diameter, 1.3 cm in thickness, and 0.02 g cm^−3^ in density) were sterile aluminum foil wrapped and sealed in zip-lock bags^9^ and installed between two stainless steel bowls of 20 cm and 30 cm diameter served as the exterior shelter with a 1.5 cm intra space between them that enable air to pass over the PUF discs^15^. Dichloromethane (DCM) and acetone were used to pre-extract all PUF discs for 48 h in the laboratory. Each Passive air sampler (PAS) was built at the deployment location to avoid contamination. PUF-PAS were installed on the rooftops of single-story buildings at the height of 5 m above ground level. Mean concentrations of gaseous-phase PAHs were collected by passive air samplers. Following the methodology stated in the previous calibration researches, the PUF standard passive air sampling rate was computed (Text S1)^49, 50^. After the sampling period, the PUFs were repaired, wrapped in aluminum foils and transferred to the laboratory and stored at − 20 °C till further analysis^10, 51^.

#### Dust sampling

Approximately 5 g of dust samples from each air sampling location were also collected after midday between 4 and 5 p.m. over the same time period as air samples. Each dust sample was made up of five separate subsamples collected from each sampling site and then combined to produce a composite sample^52^. To assemble the tiny particles, samples were gathered in stainless steel dustpans by using plastic brushes in a gentle sweeping movement. Each time new disposable dustpan and brush were used and covered with aluminum foil to minimize cross-contamination in samples^53^. Grits, hairs and organic materials were removed from the samples by subsequent sieving through 2 mm mesh (AASHTO classification). The samples were stored at − 20 °C until analysis^54^.

### Sample preparation and extraction

Air and dust samples were spiked with 50 µL of deuterated PAHs as recovery standards (Nap-d8, Phe-d10, Chry-d12, 2, 4, 5, 6-T-m-x) and separately extracted with DCM for 24 h by using Soxhlet. The samples were then extracted (in triplicate) by 30 min ultra-sonication with dichloromethane and hexane solution (1:1 v/v), followed by 1 min of vortex agitation and centrifuge for 30 min at 3500–5000 rpm at room temperature^55^. Rotary evaporation (DIAHAN Scientific WEV-1001L) was performed for volume reduction before clean-up the samples. Alumina/silica column was used to purify the samples with 8 mm internal diameter, tightly packed with 3 cm neutral alumina (3% deactivated), 50% sulfuric acid–silica, 3 cm neutral silica gel (3% deactivated) and 1 cm anhydrous Na_2_SO_4_. Later, purified sample was extracted with 1:1 DCM and hexane (by volume), blown down to a final volume of 1 mL under a moderate nitrogen flow (0.2 mL)^51^. The samples were then placed in septa vials for further examination using gas chromatography–mass spectrometer (GC–MS).

### Instrumental analysis

The samples were analyzed using GC/MS (QP2010, Shimadzu) for 16 priority PAHs (2–6 rings) in the Split Injection Mode (SIM). The injector and ion sources were both 200 °C. As the carrier gas, helium was used. The Column Flow was set to 1.6 mL/min. The oven temperature was held for 4 min at 50 °C, raised to 320 °C (held for 3 min)^54, 55^.

### Quality assurance/quality control

Throughout sampling and analysis, strict quality assurance and control procedures were followed. All the chemicals and solvents used in the current study were analytical research-grade, acquired from Sigma-Aldrich now Merck KGaA (Germany), and checked for impurities prior to use. Na_2_SO_4_ was baked for 12 h at 450 °C and stored at 120 °C till use to eliminate any organic debris. The internal and recovery standards were purchased from Chem Service, USA. All the chemicals utilized in the laboratory procedures, for example, acetone (Ace), hexane (Hex), and dichloromethane (DCM) were of the GC analytical grade. Glassware used in sample preparation was heated at 400 °C overnight and stored at 100 °C before use. A set of PAHs standards was performed daily to maintain the instrument's stability, and the instrument's fluctuation was less than 10%. Method detection limits (MDLs) of target compounds were estimated as three times the standard deviation of the mean procedural blank concentrations. Recoveries of the native analytes tested for the reference material were greater than 72% for all PAHs samples. QA/QC was performed to identify any possible laboratory contamination by conducting method blanks, standard reference material recoveries, standard spiked recoveries, and GC/MS detection limits^22^. The dilutions for standards were ranged from 0.001 to 200 μg g^−1^.

### PAHs diagnostic ratios

PAHs ratios such as Phen/Anth, Flan/Pyr, BaA/Chry and BaP/BghiP, IP/(IP + BghiP), Flu/(Flu + Pyr) and Anth/(Anth + Phen) are commonly used as tracers of PAHs emission sources^54, 56–58^. In the present study, these ratios were also determined for the source identification of PAHs.

### Human health risk assessment

#### Cancer risk assessment

The carcinogenic potential of many PAHs, particularly High Molecular Weight (HMW) PAHs, is extensively documented in the literature^59^. The benzo(a)pyrene toxic equivalency factors (TEFs) were used to estimate benzo(a)pyrene equivalent (BaPeq) or benzo(a)pyrene toxicity equivalent (BaP-TEQ) concentrations to evaluate the incremental lifetime cancer risk (ILCR) of PAHs in air and dust samples.

Equation (1) was used to calculate ILCR.

1 $${\text{BaP}} - {\text{TEQ}} = {\text{Ci}} \times {\text{TEF}}$$

BaP − TEQs was calculated by multiplying individual PAH concentration (Ci) by the WHO-recommended TEFs (toxic equivalency factors) values such as 0.001 (Nap, Ace, Acy, Fla, Phe, Flu and Pyr), 0.01 (Ant, Chr and B(ghi)P), 0.1 (B(a)a, B(b)F, B(k)F, and I(cd)P) and 1 (B(a)P and D(ah)A) established by Nisbet and LaGoy^60^ (Table 1). The computed BaP as TEQ values indicated a significant toxicity hazard linked with PAHs in air and dust samples^61^. Cancer risk from inhalation was estimated using WHO (2000) methods, and the unit risk (UR) of 8.7 × 10^−5^ (ng m^−3^) was used for a lifetime of 70 years exposure as one individual exposed to one unit BaP (1 ng m^−3^) on average. The potential cancer risk of human exposure via inhalation, ingestion, and dermal contact to air and dust-related PAHs was assessed in different age groups by an Incremental lifetime Cancer Risk (ILCR) model^53, 61^.

**Table 1:** Mean PAHs concentrations in air (pg m^−3^) during summer and winter seasons.

16 PAHs	Sampling areas																				
		Shah Jamal		UET		Ravi Road		Jail road		Tho N Baig		Misri Shah		Manawa		Gulberg		Mohlanwal		Mughalpura	
Abbr	TEFs	S	W	S	W	S	W	S	W	S	W	S	W	S	W	S	W	S	W	S	W
Naph	0.001	143.4 ± 43	144.5 ± 43	142.5 ± 42	145.8 ± 43	141.6 ± 42	145.5 ± 43	141.2 ± 42	143.2 ± 43	140.8 ± 42	144.2 ± 43	140.3 ± 42	141.5 ± 42	139.8 ± 41	142.3 ± 42	138.2 ± 41	140.5 ± 42	137.4 ± 41	138.5 ± 41	136.5 ± 41	139.4 ± 41
Ace	0.001	27.6 ± 8	28.3 ± 8	26.9 ± 8	29.1 ± 8	26.2 ± 7	28.8 ± 8	25.7 ± 7	27.4 ± 8	25.3 ± 7	27.9 ± 8	24.8 ± 7	25.6 ± 7	24.3 ± 7	26.5 ± 8	23.5 ± 7	24.5 ± 7	22.7 ± 6	23.1 ± 6	21.8 ± 6	23.5 ± 7
Acy	0.001	11.8 ± 3.5	11.8 ± 3	11.3 ± 3	12.7 ± 3	10.9 ± 3	12.2 ± 3	10.5 ± 3	10.6 ± 3	9.9 ± 3	11.3 ± 3	9.4 ± 2	9.6 ± 2	9.1 ± 2	10.4 ± 3	8.7 ± 2	8.6 ± 2	8.5 ± 2	7.2 ± 2	8.1 ± 2	7.8 ± 2
Ant	0.01	23.4 ± 7	24.6 ± 7	22.3 ± 6	26.4 ± 7	21.9 ± 6	25.5 ± 7	20.9 ± 6	22.2 ± 6	19.7 ± 5	23.4 ± 7	18.5 ± 5	20.3 ± 6	17.5 ± 5	21.3 ± 6	16.3 ± 4	19.4 ± 5	14.2 ± 4.3	16.6 ± 5	13.9 ± 4	18.7 ± 5
Flu	0.001	37.7 ± 11.3	38.9 ± 11	37.6 ± 11	39.8 ± 11	37.2 ± 11	39.3 ± 11	36.6 ± 11	37.4 ± 11	36.3 ± 10	38.4 ± 11	36.2 ± 10	35.9 ± 10	35.5 ± 10	36.6 ± 11	34.9 ± 10	35.6 ± 10	34.3 ± 10.3	34.3 ± 10	33.4 ± 10	34.9 ± 10
Phe	0.001	163.7 ± 49	163.2 ± 49	162.7 ± 48	165.8 ± 49	161.9 ± 48	164.5 ± 49	160.9 ± 48	161.5 ± 48	159.4 ± 47	162.4 ± 48	158.6 ± 47	159.6 ± 47	156.4 ± 46	160.5 ± 48	154.5 ± 46	158.5 ± 47	153.9 ± 46	156.4 ± 46	152.6 ± 45	157.3 ± 47
BaA	0.1	33.4 ± 10	34.4 ± 10	32.5 ± 9	36.6 ± 11	31.3 ± 9	35.3 ± 10	30.7 ± 9	32.4 ± 9	29.7 ± 8	33.7 ± 10	27.4 ± 8	30.7 ± 9	26.8 ± 8	31.5 ± 9	24.5 ± 7	29.3 ± 8	22.6 ± 6	27.7 ± 8	21.3 ± 6	28.6 ± 8
Chr	0.01	41.9 ± 12	42.2 ± 12	39.8 ± 11	43.9 ± 13	37.1 ± 11	43.6 ± 13	35.5 ± 10	40.3 ± 12	33.6 ± 10	41.8 ± 12	31.3 ± 9	38.9 ± 11	29.6 ± 8	39.3 ± 11	27.9 ± 8	37.7 ± 11	25.4 ± 7	34.4 ± 10	23.8 ± 7	36.6 ± 11
Fla	0.001	143.3 ± 43	143.2 ± 43	142.9 ± 42	145.1 ± 43	140.5 ± 42	144.5 ± 43	138.8 ± 41	141.1 ± 42	137.6 ± 41	142.3 ± 42	136.9 ± 41	139.5 ± 41	135.9 ± 40	140.4 ± 42	134.4 ± 40	138.4 ± 41	132.8 ± 39	136.2 ± 40	130.3 ± 39	137.5 ± 41
Pyr	0.001	112.4 ± 33	104.4 ± 31	110.5 ± 33	106.1 ± 31	108.6 ± 32	105.3 ± 31	106.8 ± 32	102.5 ± 30	104.8 ± 31	103.2 ± 31	102.7 ± 30	100.8 ± 30	100.5 ± 30	101.2 ± 30	98.9 ± 29	99.5 ± 29	96.8 ± 29	97.4 ± 29	95.3 ± 28	98.6 ± 29
BaP	1	38.3 ± 11	39.7 ± 11	37.6 ± 11	145.8 ± 43	36.9 ± 11	145.5 ± 43	36.6 ± 11	38.9 ± 11	36.2 ± 10	39.3 ± 11	35.7 ± 10	37.7 ± 11	34.9 ± 10	38.4 ± 11	33.6 ± 10	37.4 ± 11	32.9 ± 9	34.4 ± 10	31.7 ± 9	35.6 ± 10
BbF	0.1	52.8 ± 15	53.1 ± 15	52.4 ± 15	40.7 ± 12	51.8 ± 15	40.2 ± 12	50.7 ± 15	52.1 ± 15	49.8 ± 14	52.4 ± 15	48.9 ± 14	50.4 ± 15	47.9 ± 14	51.5 ± 15	46.4 ± 13	49.3 ± 14	45.4 ± 13	46.4 ± 13	43.8 ± 13	48.8 ± 14
BkF	0.1	55.9 ± 16	56.7 ± 17	55.7 ± 16	54.3 ± 16	54.8 ± 16	53.5 ± 16	54.6 ± 16	55.4 ± 16	53.9 ± 16	56.2 ± 16	53.6 ± 16	54.5 ± 16	52.9 ± 15	54.9 ± 16	52.5 ± 15	53.7 ± 16	51.4 ± 15	51.3 ± 15	50.4 ± 15	52.3 ± 15
DahA	1	59.2 ± 17	57.5 ± 17	58.4 ± 17	57.9 ± 17	56.7 ± 17	57.4 ± 17	54.6 ± 16	53.4 ± 16	52.6 ± 15	55.3 ± 16	50.8 ± 15	50.2 ± 15	49.7 ± 14	52.8 ± 15	47.6 ± 14	49.8 ± 14	45.9 ± 13	45.5 ± 13	45.3 ± 13	47.6 ± 14
IP	0.1	36.2 ± 10	36.5 ± 11	35.9 ± 10	61.3 ± 18	34.8 ± 10	59.3 ± 17	33.6 ± 10	34.3 ± 10	32.5 ± 9	35.5 ± 10	31.9 ± 9	32.5 ± 9	30.7 ± 9	33.5 ± 10	29.9 ± 9	31.4 ± 9	28.3 ± 8	27.4 ± 8	27.4 ± 8	29.3 ± 8
BghiP	0.01	29.9 ± 9	29.6 ± 8	29.2 ± 8	38.8 ± 11	28.4 ± 8	37.4 ± 11	28.2 ± 8	28.3 ± 8	27.5 ± 8	29.3 ± 8	27.1 ± 8	27.8 ± 8	26.6 ± 8	28.1 ± 8	26.3 ± 7	27.5 ± 8	25.4 ± 7	26.5 ± 8	25.1 ± 7	26.8 ± 8
∑_16_PAH		1010.9 ± 303	1008.6 ± 302	998.2 ± 299	1035.8 ± 310	980.6 ± 294	1023.4 ± 307	965.9 ± 289	981 ± 294	949.6 ± 284	996.6 ± 299	934.1 ± 280	955.5 ± 286	918.1 ± 275	969.2 ± 290	898.1 ± 269	941.1 ± 282	877.9 ± 263	903.3 ± 271	860.7 ± 258	923.3 ± 277
∑_LMPAH_		407.6 ± 122	411.3 ± 123	403.3 ± 120.9	419.6 ± 125	399.7 ± 119	415.8 ± 124	395.8 ± 118	402.3 ± 120	391.4 ± 117	407.6 ± 122	387.8 ± 116.3	392.5 ± 117	382.6 ± 114	397.6 ± 119	376.1 ± 112	387.1 ± 116	371.0 ± 111	376.1 ± 112.8	366.3 ± 109	381.6 ± 114
∑_HMPAH_		603.3 ± 181	597.3 ± 179	594.9 ± 178	616.2 ± 184	580.9 ± 174	607.6 ± 182	570.1 ± 171	578.7 ± 173	558.2 ± 167	589 ± 176	546.3 ± 163	563.0 ± 168	535.5 ± 160	571.6 ± 171	522.0 ± 156	554 ± 166	506.9 ± 152	527.2 ± 158	494.4 ± 148	541.7 ± 162
∑C_7PAH_		358.9 ± 107	354.4 ± 106	353.8 ± 106	365.9 ± 109	346.6 ± 104	360.2 ± 108	341.2 ± 102	343.9 ± 103	334.4 ± 100	349.6 ± 104	327.3 ± 98	334.4 ± 100	320.3 ± 96	339.1 ± 101	312.1 ± 93	328.1 ± 98	302.8 ± 90	311.1 ± 93	295.0 ± 88	320 ± 96
Bap		116.9	116.9	115.2	122.4	112.4	119.5	109.6	111.3	106.8	114.0	104.1	106.2	101.8	109.8	97.83	105.0	94.8	96.5	92.5	100.5

∑L_6_PAH = less than four aromatic rings PAHs (i.e. Naph, Ace, Acy, Ant, Flu, Phe).

∑H_10_PAHs = four or more rings PAHs (i.e. BaA, Chr, Fla, Pyr, BaP, BbF, BkF, DahA, IP, BghiP).

∑C_7_PAHs = carcinogenic PAHs (i.e. BaA, BaP, BbF, BkF, Chry, DahA and IP).

Equation (2)–(4) were employed to calculate cancer risk via various exposure routes.

2 $${\text{ILCR}}_{{{\text{Ingestion}}}} {:}\;\frac{{BaP - TEQ \times \left( {CSF_{Ingestion} \times \sqrt[3]{{\left( {\frac{BW}{{70}}} \right)}}} \right) \times IR_{Ingestion } \times EF \times ED}}{{BW \times AT \times 10^{6} }}$$

3 $${\text{ILCR}}_{{{\text{Dermal}}}} {:}\;\frac{{BaP - TEQ \times \left( {CSF_{Dermal} \times \sqrt[3]{{\left( {\frac{BW}{{70}}} \right)}}} \right) \times SA \times AF \times ABS \times EF \times ED}}{BW \times AT \times PEF}$$

4 $${\text{ILCR}}_{{{\text{Inhalation}}}} {:}\;\frac{{BaP - TEQ \times \left( {CSF_{Inhalation} \times \sqrt[3]{{\left( {\frac{BW}{{70}}} \right)}}} \right) \times IR_{Inhalation } \times EF \times ED}}{BW \times AT \times PEF}$$

where BaP − TEQ is the total of converted PAHs levels based on toxic equivalents of BaP calculated by multiplying each PAH concentration (c_i_) with the toxic equivalency factor (TEF). CSF is carcinogenic slope factor (mg kg^−1^ day^−1^), BW is body weight (kg), AT is the average life span (years), EF is the exposure frequency (day year^−1^), ED is the exposure duration (years), IR_Inhalation_ is the inhalation rate (m^3^ day^−1^), IR_Ingestion_ is the soil intake rate (mg day^−1^), SA is the dermal surface exposure (cm^2^), AF is the dermal adherence factor (mg cm^2^ h^−1^), ABS is the dermal adsorption fraction, and PEF is particle emission factor (m^3^ kg^−1^)^62^. CSF_Ingestion_, CSF_Dermal_ and CSF_Inhalation_ of BaP were addressed as 7.3, 25, and 3.85 (mg kg^−1^ day^−1^), respectively, determined by the cancer-causing ability of BaP^63^. All of the parameters included in this model were based on the United States Environmental Protection Agency's (US EPA) Risk Assessment Guidance and associated publications^64, 65^. Values of the parameters used in the above equations are presented in Table S1 (“Supplementary material S1”).

#### Non-carcinogenic risk

The non-cancer risk assessment of PAHs is essentially examining the association between PAHs dose and unfavourable health effects. It primarily consisted of estimating PAHs exposure dose using various environmental matrices (in this study, dust and air), exposure pathways and exposure frequency^66^. Although, other parameters such as age and body weight may influence the frequency and duration of exposure. The non-cancer risk of PAHs was evaluated in this study for five age groups based on ingestion and inhalation pathways using the Eqs. (5) and (6) to determine the health risk of daily oral and breathing intake of PAHs from dust and air, respectively^10, 14, 67^.

5 $${\text{EDI}}_{{{\text{Ingestion}}}} = {\text{C}}_{{{\text{dust}}}} \cdot {\text{f}}_{{1}} /{\text{M}}_{{1}}$$

The equation to determine the health risk of breathing intake^10^:

6 $${\text{EDI}}_{{{\text{Inhalation}}}} = {\text{C}}_{{{\text{air}}}} \cdot {\text{f}}_{{2}} /{\text{M}}_{{1}}$$

## Results and discussion

### Distribution and levels of PAHs in air

The ∑_16_PAHs mean concentrations (pg m^−3^) in air during summer and winter seasons from the study area have shown in Table 1. Lowest to highest mean concentrations of ∑_16_PAHs ranged from 258.2 ± 860.7 to 303.3 ± 1010.9 (pg m^−3^) and 903.3 ± 271.0 to 1035.8 ± 310.7 (pg m^−3^) in summer and winter, respectively. The mean concentrations of lower molecular weights PAHs (∑_LMPAHs_) (and high molecular weight PAHs (∑_HMPAHs_) ranged from 366.3 ± 109.9 to 407.6 ± 122.3 and 494.4 ± 148.3 to 603.3 ± 181.0 (pg m^−3^) in summer and 376.1 ± 112.8 to 419.6 ± 125.9 and 527.2 ± 158.2 to 616.2 ± 184.9 (pg m^−3^) in winter seasons of the study period. The mean carcinogenic PAHs (∑C_7_PAHs) concentrations were ranged from 295.0 ± 88.5 to 358.9 ± 107.7 and 311.1 ± 93.3 to 365.9 ± 109.8 (pg m^−3^) in summer and winter, respectively. Among all the PAHs studied, Phe, Nap, and Pyr were found at significant levels in air. The concentration range of studied PAHs for summer and winter in air is mentioned in Tables S2 and S3, respectively.

The current study's findings showed a substantially lower range of ∑_16_ PAHs than earlier PAHs studies conducted in contaminated places, such as Khatmandu in Nepal (155,000 pg m^−3^)^68^, Tehran in Iran (57,000 pg m^−3^)^58^, Ningbo in China (46,000 pg m^−3^)^69^ and Xian in China (116,000 pg m^−3^)^70^. Due to the meteorological and geographical variations, the composition and concentration of PAHs vary in Lahore from the other urban environments of the world. The findings of the current study agreed with a study reported from twin cities of Pakistan (2132 pg m^−3^)^10^ and Paris in France (1000 pg m^−3^)^71^ (Table S6).

### Levels and distribution of PAHs in road dust

Mean concentrations of ∑_16_PAHs in road dust ranged from 245.7 ± 818.9 to 283.6 ± 945.2 (ng g^−1^) and 256.8 ± 853.4 to 89.0 ± 963.4 (ng g^−1^) in summer and winter seasons, respectively (Table 2). The mean concentrations of ∑_LMPAHs_ and ∑_HMPAHs_ were ranged between 348.8 ± 104.6 and 387.3 ± 116.2 (ng g^−1^) and 470.1 ± 141.0 to 550.2 ± 165.1 (ng g^−1^) in summer while 359.8 ± 108.9 to 397.6 ± 119.3 (ng g^−1^) and 488.9 ± 146.7 to 565.8 ± 169.7 (ng g^−1^) during winter seasons. The mean ∑C_7_PAHs concentrations were ranged from 282.6 ± 84.8 to 331.3 ± 99.4 (ng g^−1^) and 285.2 ± 85.6 to 331.8 ± 99.5 (ng g^−1^) in summer and winter, respectively. Phe, Nap and Fla levels showed the highest concentrations in both seasons for dust samples. However, PAHs identified in dust samples showed higher winter concentrations than summer during the study period. The concentrations range of PAHs parameters for summer and winter dust are mentioned in Tables S4 and S5, respectively.

**Table 2:** Mean PAHs concentrations in dust (ng g^−1^) during summer and winter seasons.

16 PAHs	Sampling areas																			
	Shah Jamal		UET		Ravi Road		Jail road		Tho N Baig		Misri Shah		Manawa		Gulberg		Mohlanwal		Mughalpura	
Abbr	s	W	s	W	s	w	s	w	s	w	s	w	s	w	s	w	s	w	s	w
Naph	133.2 ± 40	138.4 ± 41	132.7 ± 39	139.3 ± 41	134.3 ± 40	136.7 ± 41	132.0 ± 39	135.8 ± 41	129.8 ± 38	137.2 ± 41	126.8 ± 38	134.3 ± 41	130.9 ± 39	133.1 ± 40	127.0 ± 38	132.9 ± 40	128.7 ± 38	130.4 ± 39	127.6 ± 38	129.5 ± 39
Ace	22.9 ± 6	25.1 ± 7	21.5 ± 6	25.5 ± 7	23.2 ± 7	24.6 ± 7	21.1 ± 6	24.2 ± 7	19.2 ± 5	24.9 ± 7	18.0 ± 5	23.4 ± 7	19.7 ± 5	23.1 ± 6	18.2 ± 5	22.7 ± 6	19.0 ± 5	21.8 ± 6	18.5 ± 5	21.1 ± 6
Acy	8.6 ± 2	9.3 ± 2	8.1 ± 2	9.5 ± 2	8.9 ± 2	8.8 ± 2	7.5 ± 2	8.5 ± 2	7.1 ± 2	9.1 ± 2	6.0 ± 1	8.1 ± 2	7.3 ± 2	7.6 ± 2	6.3 ± 1	7.3 ± 2	6.9 ± 2	7.2 ± 2	6.5 ± 2	6.5 ± 1
Ant	22.3 ± 6	23.3 ± 7	21.9 ± 6	23.8 ± 7	22.8 ± 6	20.9 ± 6	21.4 ± 6	20.5 ± 6.2	20.8 ± 6.2	21.2 ± 6	16.2 ± 4	19.9 ± 6	21.0 ± 6	18.6 ± 5	17.2 ± 5	17.7 ± 5	19.5 ± 5	16.8 ± 5	18.3 ± 5	15.8 ± 4
Flu	33.8 ± 10	36.3 ± 10	34.9 ± 10	36.8 ± 11	34.3 ± 10	35.5 ± 10	33.6 ± 10	35.1 ± 10	31.2 ± 9	35.9 ± 10	29.3 ± 8	34.8 ± 10	32.3 ± 9	34.1 ± 10	30.9 ± 9	33.8 ± 10	30.5 ± 9	33.5 ± 10	29.5 ± 8	33.1 ± 9
Phe	162.4 ± 48	161.2 ± 48	161.9 ± 48	162.7 ± 48	163.8 ± 49	159.2 ± 47	160.9 ± 48	158.6 ± 47	157.6 ± 47	160.4 ± 48	152.5 ± 45	157.5 ± 47	159.4 ± 47	156.7 ± 47	153.9 ± 46	155.8 ± 46	156.5 ± 47	154.8 ± 46	155.4 ± 46	153.8 ± 46
BaA	33.5 ± 10	33.3 ± 10	32.6 ± 9	33.8 ± 10	33.9 ± 10	32.9 ± 9	32.9 ± 9	32.6 ± 9	31.4 ± 9	33.1 ± 9	29.3 ± 8	32.4 ± 9	32.7 ± 9	31.5 ± 9	29.7 ± 8	31.3 ± 9	30.8 ± 9	29.6 ± 8	30.5 ± 9	29.3 ± 8
Chr	36.8 ± 11	39.3 ± 11	35.1 ± 10	39.9 ± 12	37.3 ± 11	38.1 ± 11	32.9 ± 9	37.3 ± 11	32.3 ± 9	38.4 ± 11	29.0 ± 8	36.4 ± 10	33.6 ± 10	35.7 ± 10.7	29.4 ± 8	34.8 ± 10	31.9 ± 9	34.2 ± 10	30.9 ± 9	33.5 ± 10
Fla	137.6 ± 41	139.5 ± 41	136.5 ± 41	141.7 ± 42	138.5 ± 41	135.7 ± 40	135.8 ± 40	133.5 ± 40	130.9 ± 39	137.3 ± 41	122.8 ± 36	131.3 ± 39	133.6 ± 40	129.6 ± 38	124.8 ± 37	127.7 ± 38	128.9 ± 38	125.9 ± 37	126.4 ± 37	124.8 ± 37
Pyr	93.5 ± 28	96.3 ± 28	92.6 ± 27	97.7 ± 29	94.9 ± 28	94.4 ± 28	91.8 ± 27	93.6 ± 28	89.7 ± 26	95.3 ± 28	85.3 ± 25	92.5 ± 27	90.8 ± 27	91.8 ± 27	86.8 ± 26	90.9 ± 27	88.5 ± 26	89.3 ± 26	87.9 ± 26	88.7 ± 26
BaP	48.6 ± 14	37.5 ± 11	47.9 ± 14	37.9 ± 11	48.9 ± 14	36.6 ± 11	47.6 ± 14	36.3 ± 10	45.7 ± 13	37.3 ± 11	42.1 ± 12	35.1 ± 10	46.9 ± 14	34.7 ± 10	43.9 ± 13	33.6 ± 10	45.1 ± 13	32.8 ± 9	44.6 ± 13	51.9 ± 15
BbF	48.4 ± 14.5	49.4 ± 14.8	47.8 ± 14.3	50.8 ± 15.2	48.9 ± 14	48.5 ± 14.6	47.3 ± 14	47.5 ± 14	45.9 ± 13	48.9 ± 14	42.3 ± 12	47.4 ± 14	46.8 ± 14	46.8 ± 14	43.5 ± 13	45.8 ± 13.7	45.2 ± 13	44.5 ± 13	44.4 ± 13	43.9 ± 13
BkF	48.7 ± 14	50.3 ± 15	48.1 ± 14	51.7 ± 15	49.2 ± 14	48.6 ± 14	47.6 ± 14	47.7 ± 14	45.6 ± 13	49.4 ± 14	41.8 ± 12	46.8 ± 14	46.9 ± 14	45.6 ± 13	42.4 ± 12	44.8 ± 13	44.9 ± 13	43.8 ± 13.1	43.5 ± 13	41.8 ± 12
DahA	49.4 ± 14	51.2 ± 15	47.7 ± 14	52.4 ± 15	50.8 ± 15	49.6 ± 14	46.6 ± 14	48.5 ± 14	42.8 ± 12	50.3 ± 15	35.7 ± 10	47.7 ± 14	44.6 ± 13	46.7 ± 14	38.9 ± 11	45.9 ± 13	40.7 ± 12	43.6 ± 13	39.6 ± 11	42.9 ± 12
IP	30.2 ± 9	32.4 ± 9	29.2 ± 8	33.4 ± 10	31.8 ± 9	29.6 ± 8	28.8 ± 8	28.5 ± 8	26.6 ± 8	30.4 ± 9	22.3 ± 6	27.8 ± 8	27.8 ± 8	26.8 ± 8	23.6 ± 7	25.9 ± 7	25.4 ± 7	23.8 ± 7	24.1 ± 7	21.7 ± 6
BghiP	23.5 ± 7	26.3 ± 7	22.6 ± if6	26.5 ± 8	23.7 ± 7	25.2 ± 7	22.1 ± 6	24.8 ± 7	21.7 ± 6	25.7 ± 7	19.5 ± 5	24.3 ± 7	21.9 ± 6	23.5 ± 7	20.1 ± 6	22.9 ± 6	21.3 ± 6	21.4 ± 6	20.5 ± 6	20.5 ± 6
∑16PAH	933.4 ± 280	949.1 ± 284	921.1 ± 276	963.4 ± 289	945.2 ± 283	924.9 ± 278	911.5 ± 273	913.0 ± 274	878.3 ± 263	934.8 ± 281	818.9 ± 245	899.7 ± 270	896.2 ± 268	885.9 ± 266	836.6 ± 251	873.8 ± 262	863.8 ± 259	853.4 ± 256	848.2 ± 254	858.8 ± 258
∑_L6MPAH_	383.2 ± 115	393.6 ± 118	381.0 ± 114	397.6 ± 119	387.3 ± 116	385.7 ± 116	376.5 ± 113	382.7 ± 115	365.7 ± 109	388.7 ± 117	348.8 ± 104	378.0 ± 114	370.6 ± 111	373.2 ± 112	353.5 ± 106	370.2 ± 111	361.1 ± 108	364.5 ± 110	355.8 ± 106	359.8 ± 108
∑_H10MPAH_	550.2 ± 165	555.5 ± 166	540.1 ± 162	565.8 ± 169	557.9 ± 167	539.2 ± 161	535.0 ± 160	530.3 ± 159	512.6 ± 153	546.1 ± 163	470.1 ± 141	521.7 ± 156	525.6 ± 157	512.7 ± 153	483.1 ± 144	503.6 ± 151	502.7 ± 150	488.9 ± 146	492.4 ± 147	499 ± 149
∑C_7PAH_	326.4 ± 97	325.5 ± 97	320.8 ± 96	331.8 ± 99	331.3 ± 99	315.8 ± 94	318.1 ± 95	311.0 ± 93	306.6 ± 92	320.1 ± 96	282.6 ± 84	306.3 ± 91	313.8 ± 94	300.7 ± 90	290.0 ± 87	295.2 ± 88	301.2 ± 90	285.2 ± 85	295.5 ± 88	297.8 ± 89
Bap	115.5	106.7	112.8	108.8	117.5	103.6	111.2	101.8	104.8	105.2	92.6	99.6	108.3	97.8	97.9	95.6	101.7	109.7	99.7	91.9

Findings of the present study have observed higher mean concentrations from the previous study conducted in Pakistan, i.e. Rawalpindi and Islamabad (89.8 ng g^−1^ d.w.)^10^ and showed similar findings with Chung Khurd, Lahore (882 ng g^−1^ d.w.)^72^. In comparison with other international cities, the current study has shown higher mean concentrations of Σ_16_PAHs of dust compared from Karaj, Iran (624 ng g^−1^ d.w.)^73^ and lower than Lanzhou city, China (3900 ng g^−1^ d.w.)^65^, Sydney, Australia (2910 ng g^−1^ d.w.)^74^, Tianjin, China (7993.3 ng g^−1^ d.w.)^14^, New Delhi, India (1100 ng g^−1^ d.w.), and Mashhad, Iran (2183.5 ng g^−1^ d.w.)^75^. Furthermore, the current study results depicted similar findings to the study conducted in PAHs of dust in Ulsan, Korea (960 ng g^−1^ d.w.)^76^ (Table S7).

### Spatial distribution pattern of PAHs in Lahore

Lahore, the country's biggest traffic hub, had a tremendous influx of traffic every day, causing traffic congestion and eventually increasing vehicular emissions^28^. Furthermore, poor car engines maintenance and fuel quality had a substantial role in PAHs emissions^77^. The concentrations of PAHs in Lahore city air and dust varied with seasons. The greatest PAHs concentrations in the air were detected in Shah Jamal and UET, followed by Ravi road, where traffic pollution is to blame for the increasing PAHs levels in these areas. Among all study sites, the dust samples taken from UET road had the highest PAH concentrations. It is the oldest and largest high-traffic area globally, with heavily inhabited streets on both sides of a 2-km span^78^. Due to the high population density in the city, the sites are subjected to considerable traffic and domestic heating^77^. The study's findings revealed that the PAHs distribution in the dust around Lahore is not uniform but rather the result of a number of contributing elements like heavy traffic and distance from industry.

### Profile of PAHs in air and dust samples

**Figure 2:**
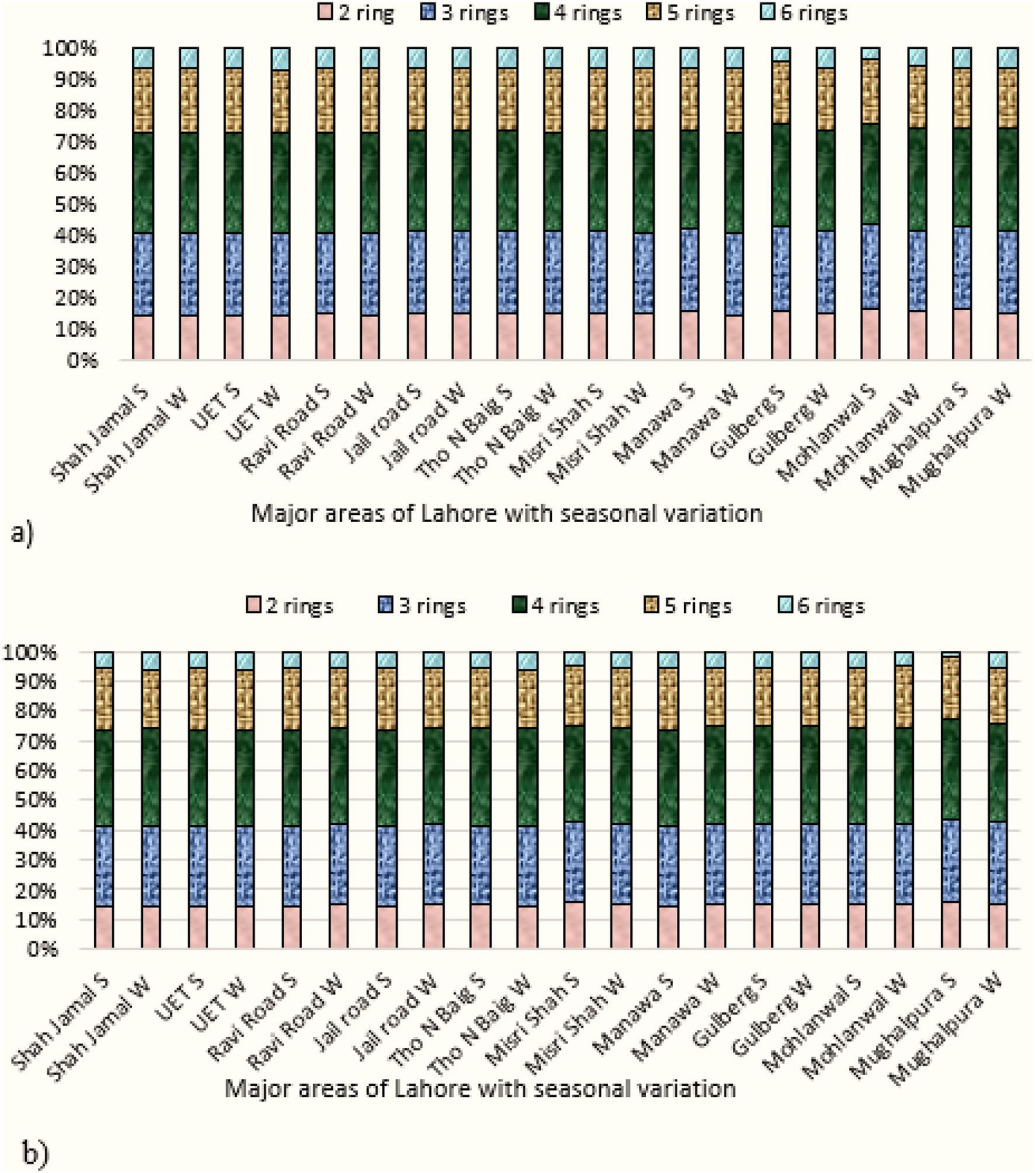
Spatio-Temporal variation in ring wise composition of PAHs in (**a**) air and (**b**) dust samples of Lahore city, Pakistan.

A significant variation was detected among PAHs concentrations in air, and dust samples. The PAHs profile distributions was in order of 4 rings (air: 31%, dust: 32%) > 3 rings (air: 26%, dust: 27%) > 5 rings (air: 19%, dust: 20%) > 2 rings (air: 14%, dust: 13%) and > 6 rings (air: 10%, dust: 8%) PAHs, respectively. The most dominant PAHs in this investigation were 3 rings (Acy, Flu, Phe, and Ant) and 4 rings (Pyr, BAA, and CHR) with a cumulative percentage contribution of 55% in air and 57% in dust (Fig. 2a,b). This compositional pattern is similar to previous studies showing 3 and 4 ring PAHs as the main contributors of PAHs^2, 79, 80^. The observed trend can be explained by considering the physicochemical characteristics of PAHs and their nearness to their origins^79^. In the present study, 4 rings PAHs were found in relatively higher concentrations, representing the pyrogenic origin and biomass fuel combustion, followed by 3 rings PAHs, suggesting the markers of petroleum-derived residues^22, 41^. The dominance of HMW (4, 5 and 6 rings) PAHs was similar to the results conducted by Najmeddin and Keshavarzi^55^ in Ahvaz city where HMW PAHs (dust: 68.8%) showed higher concentrations. The amount of PAHs in the atmosphere is also influenced by several variables such as emission sources and meteorological characteristics, including rainfall, temperature, wind speed and direction, resulting in seasonal variation in PAHs levels^81^. Higher PAHs emissions in the winter season are due to increased combustion of biomass and fossil fuels for household heating and the usual increase in primary pollutants in the colder months due to poorer dispersion conditions and lower atmospheric temperature compared to the summer (high-temperature) seasons^82, 83^. Thus, the overall results of the PAHs profile revealed not only biomass combustion but also vehicular emission as the source of PAHs deposition in Lahore because all of the selected sampling sites are known for traffic pollution due to the high rate of daily traffic flow on the roadways^1, 4^.

### PAHs isomeric ratio in air and dust

In the present study, PAHs ratios such as Phen/Anth, Flan/Pyr, BaA/Chry, and BaP/BghiP, IP/(IP + BghiP), Flu/(Flu + Pyr) and Anth/(Anth + Phen) were determined to predict the origins of PAHs, which possesses a significant hazard to the population^46, 54, 58^. PAHs generated from various sources have considerable diverse compositional patterns^84, 85^. Ant/(Ant + Phe) ratios less than 0.1 suggest a petroleum source, whereas ratios greater than 0.1 implies that combustion is dominant^65, 86^. In the present study, Ant/(Ant + Phe) ratios ranged from (0.05–0.15; 0.05–0.13), indicating the dominance of petroleum sources. Furthermore, a Flu/(Flu + Pyr) ratio of 0.4 suggests a petroleum input source, 0.4–0.5 for liquid fossil fuel and crude oil combustion, > 0.5 for biomass and coal combustion and the ratio of InP/(InP + BP) < 0.20 indicates a petroleum source, > 0.50 for the biomass and coal combustion, and 0.20–0.50 for liquid fossil fuel combustion^86^.

**Figure 3:**
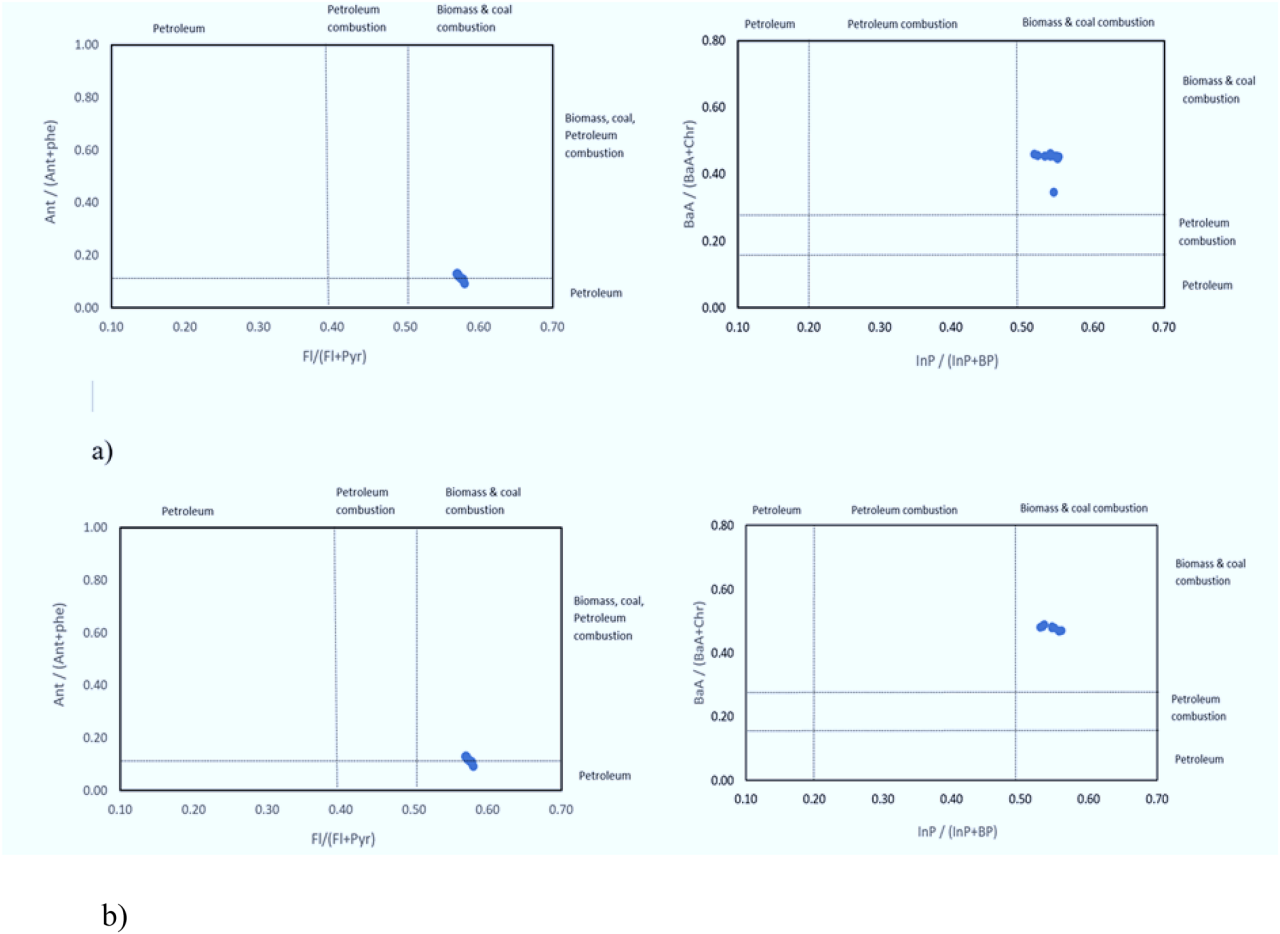
Cross plots for isomeric ratios of (**a**) Ant/(Ant + Phe) and Fl/(Fl + Pyr)in air, and (**b**) BaA/(BaA + Chr) and Inp/(Inp + BP) in dust of Lahore city, Pakistan.

**Figure 4:**
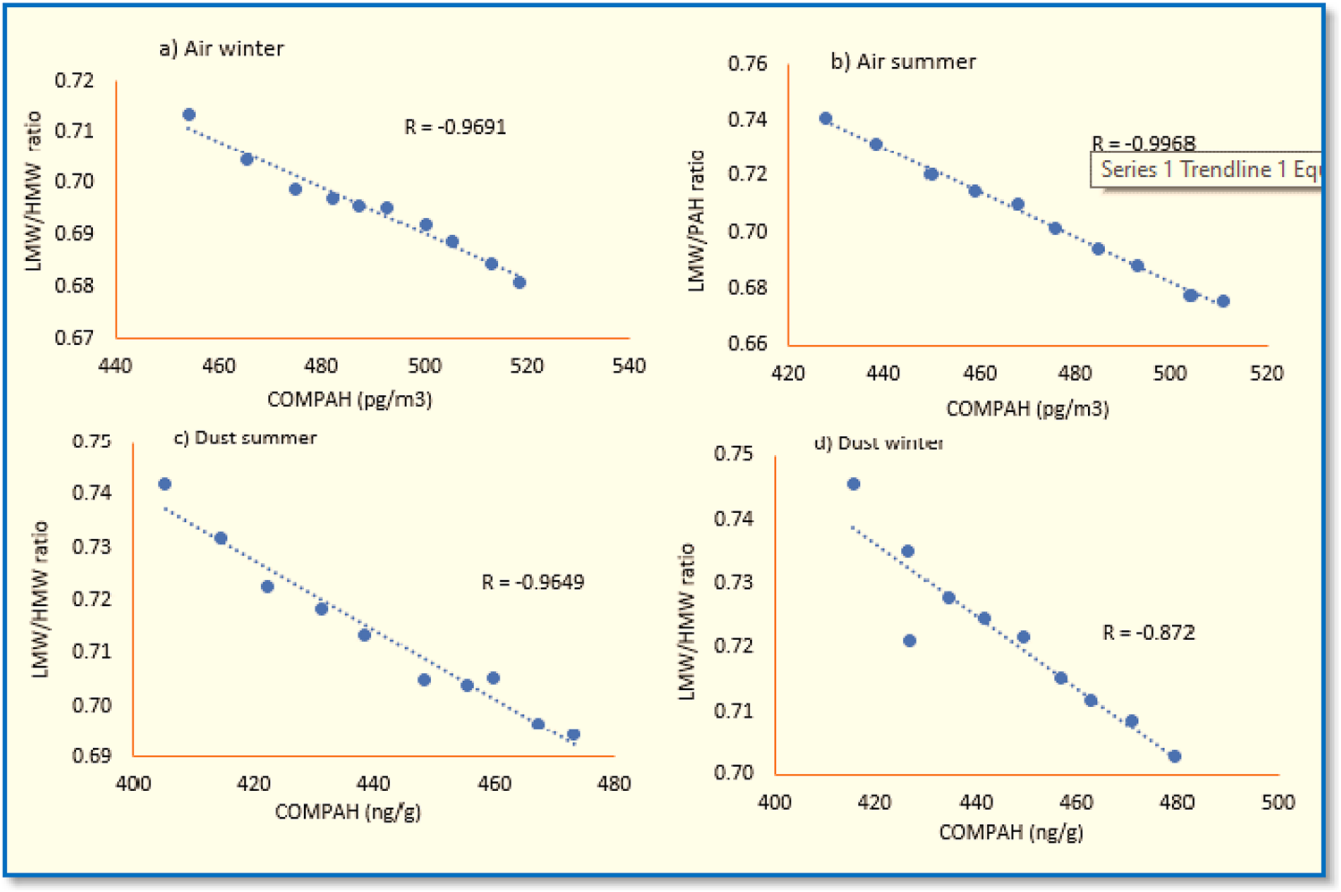
Spearmen correlations between combustion PAHs (COMPAHs) and LMW/HMW ratio identified (**a**, **b**) in air samples and (**c**, **d**) dust samples during summer and winter seasons, respectively.

In the current study, ratios of Flu/(Flu + Pyr) (0.51–0.58; 0.52–0.59) and InP/(InP + BP) (0.51–0.58; 0.52–0.59) showed the major contribution from biomass and coal combustion^86^. While the ratio of BaA/(BaA + Chr) < 0.2 stands for petroleum, 0.2–0.35 for liquid fossil fuel, vehicle and crude oil, and > 0.35 for combustion of coal, grass and wood^86^. As the ratio of BaA/(BaA + Chr) in this study ranged from (0.31–0.49; 0.49–0.53), representing vehicular emission and grass and wood combustion are highlighted as key sources of PAHs in air and dust of Lahore city (Fig. 3a,b). Furthermore, the LMW/HMW PAHs ratio was employed to estimate the extent of burning activities and pyrogenic and petrogenic sources of PAHs^10^. The current study's findings revealed that petroleum combustion was a prominent PAHs contributor since LMW/HMW PAHs ratio was less than 1 during both summer and winter seasons in air (0.68–0.74) and dust samples (0.69–0.75), respectively. It is further supported by the results of Spearmen Correlations, showing the negative relationship between LMW/HMW PAHs ratio for air (summer: R2 = 0.9691; winter: R2 = 0.9968) (Fig. 4a,b) and dust (summer: R2 = 0.9649; winter: R2 = 0.872) (Fig. 4c,d). Current results are consistent with the findings reported by He et al.^84^ from Nanjing, China, where fossil fuel burning was recognized as the primary source of PAHs and Hamid et al.^10^, where the indoor and outdoor air PAHs relation with fuel combustion in Pakistan's twin cities was found (Rawalpindi and Islamabad).

### Health risk assessment of PAHs in air and dust

#### Carcinogenic risk

The total BaPeq of ∑16PAHs in air and dust samples ranged from 92.5 to 122.4 pg m^−3^ and 91.9–117.5 ng g^−1^ during the study period, respectively (Tables 1 and 2). The current study's findings were comparable to the PAHs levels in street dust of Nanjing, China, from 25.9 to 90.8 (ng g^−1^)^84^. The cancer risk from various routes of exposure was found to be in the order: dermal contact > ingestion > inhalation. Total ILCR estimates utilizing maximum concentrations showed a possible cancer risk for persons residing in sampling areas. The total ILCR values in air samples were children (summer: 9.61E − 02, winter: 2.09E − 02) and adults (summer: 1.45E − 01, winter: 3.14E − 02) and in dust samples of selected areas, children summer: 9.16E − 03, winter: 8.80E − 03 and adults summer: 1.38E − 02, winter: 1.33E − 02 during the study period (Table 3). According to the present study, both ingestion and dermal contact increased cancer with the magnitude of 1E − 02 and 1E − 03 in air and dust samples, respectively, contributing significantly to cancer risk in children and adults (Table 3).

**Table 3:** Total risk (∑ILCR) in ∑_16_ PAHs in air and dust samples of Lahore City, Pakistan.

Seasons	Age group	Gender	Ingestion	Dermal	Inhalation	Total risk	Mean total risk
**Air samples**							
Summer	Child	Male	4.23E − 02	5.27E − 02	1.64E − 06	9.50E − 02	9.61E − 02
		Female	4.33E − 02	5.39E − 02	1.68E − 06	9.72E − 02	
	Adults	Male	5.06E − 02	8.98E − 02	3.92E − 06	1.40E − 01	1.45E − 01
		Female	5.38E−02	9.55E − 02	4.17E − 06	1.49E − 01	
Winter	Child	Male	9.18E − 03	1.14E − 02	3.56E − 07	2.06E − 02	2.09E − 02
		Female	9.39E − 03	1.17E − 02	3.64E − 07	2.11E − 02	
	Adults	Male	1.10E − 02	1.95E − 02	8.51E − 07	3.05E − 02	3.14E − 02
		Female	1.17E − 02	2.07E − 02	9.05E − 07	3.24E − 02	
**Dust samples**							
Summer	Child	Male	4.03E − 03	5.02E − 03	1.56E − 07	9.05E − 03	9.16E − 03
		Female	4.12E − 03	5.14E − 03	1.60E − 07	9.26E − 03	
	Adults	Male	4.82E − 03	8.56E − 03	3.74E − 07	1.34E − 02	1.38E − 02
		Female	5.12E − 03	9.10E − 03	3.97E − 07	1.42E − 02	
Winter	Child	Male	3.87E − 03	4.83E − 03	1.50E − 07	8.70E − 03	8.80E − 03
		Female	3.96E − 03	4.94E − 03	1.54E − 07	8.90E − 03	
	Adults	Male	4.63E − 03	8.23E − 03	3.59E − 07	1.29E − 02	1.33E − 02
		Female	4.92E − 03	8.75E − 03	3.82E − 07	1.37E − 02	

According to epidemiological research, long-lasting PAHs exposure has been linked to increased skin, lung and gastrointestinal malignancies^5, 22, 54^. For adults, skin contact was the most common exposure route because PAHs enter in the body very easily by the dermal contact with soil, contaminated water, soot, tar or by applying few oils on the body that contain high levels of PAHs, which resulted in a substantially increased risk, followed by the ingestion pathway^51^. An ILCR between E − 06 and E − 04 specified a potential carcinogenic threat, whereas an ILCR between E − 04 and E − 042 indicated a high-potential health hazard^4^. In the current research, ILCRs of total cancer risk for both children and adults were greater than the baseline tolerable risk, suggesting a high cancer risk. According to the findings, dust-borne PAHs pose a risk to local inhabitants in Lahore, comparable to research done by Jiang et al.^85^, where ILCR was higher from 5.34 E − 05 to 4.50 E − 04.

#### Non-carcinogenic risk

PAHs can induce health hazards that are not always linked to cancer but can show significant consequences for non-cancerous health risks such as asthma, heart problems, acute lung dysfunction, jaundice, kidney and liver failure^51, 52^. Microbial diversity and metabolic profiles may serve as response markers to PAHs exposure in children with asthma. Inhaling PAHs causes hypersensitivity of immunoglobulin E (IgE) substance associated with increasing the asthma emergency department visits in all age groups^87, 88^. PAHs, such as Naphthalene, are extremely carcinogenic, can induce kidney and liver damage, cause redness and irritation of skin through dermal contact and cause red blood cell destruction when breathed. Primarily the industrial workers exposed to PAHs and other chemicals were shown to have an elevated risk of skin, lung, bladder, and gastrointestinal malignancies in many studies^89^. PAHs metabolites are related to increased risk of atherosclerotic cardiovascular disease (ASCVD) in the general population, changing the heart rate variability (HRV), an early marker of cardiac autonomic imbalance^90^. Chronic exposure to PAHs induced oxidative stress, involved in the development of diabetes^91, 92^. Additionally, additive effect of reduced lung function and urinary OH-PAHs on diabetes was also found^93^.

**Figure 5:**
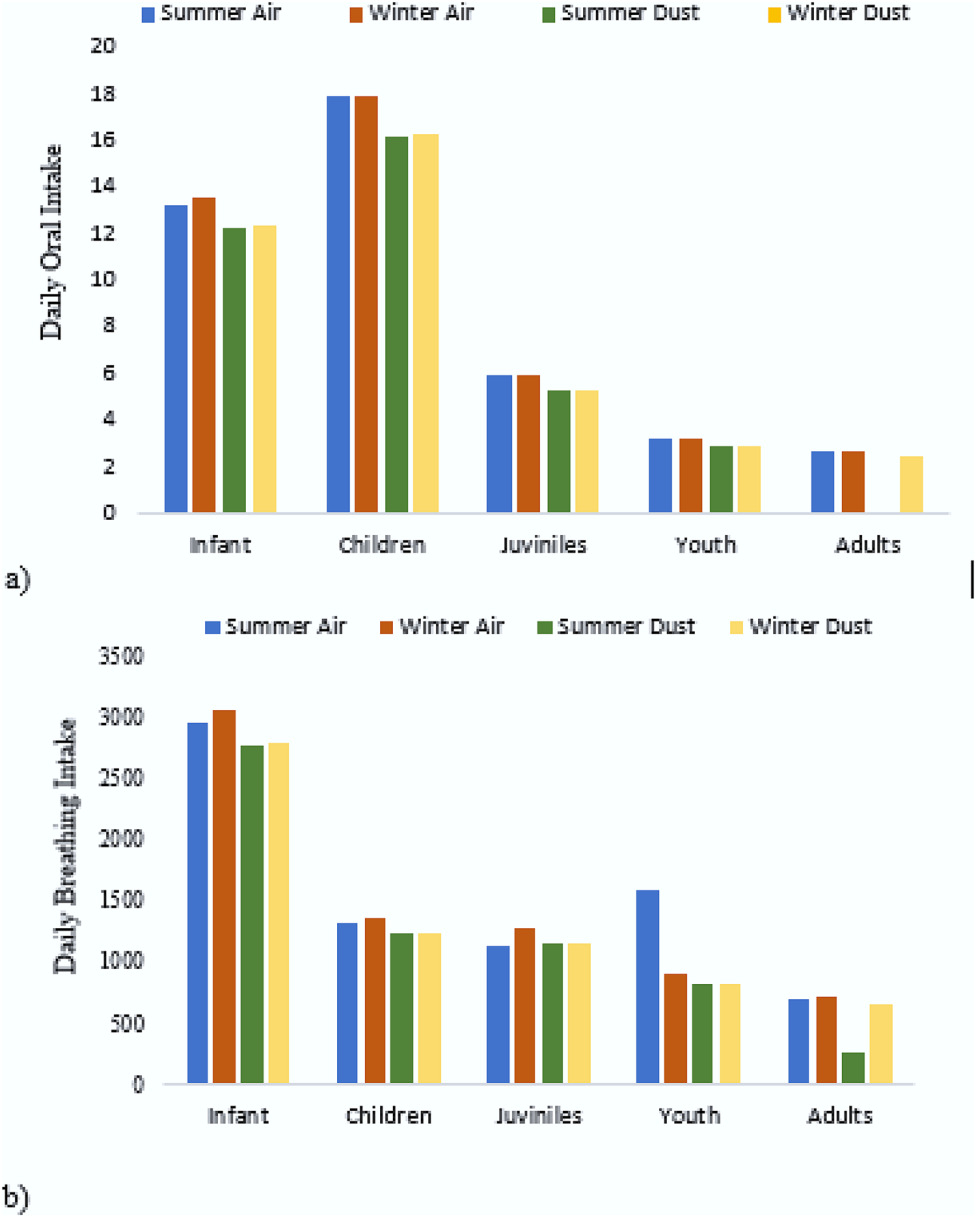
EDI through (**a**) ingestion and (**b**) breathing of the air and dust samples during summer and winter season from Lahore City, Pakistan.

Total estimated daily intake (EDI) PAHs of oral intake in air samples ranged from 2.7 to 17.9 ng kg^−1^ day^−1^ for two age cohorts in the air samples, i.e. children: 17.9 ng kg^−1^ day^−1^ and adults: 2.7 ng kg^−1^ day^−1^ in summer seasons and a similar trend was observed during winter seasons, exhibiting relative higher winter EDI in children than adults. The oral PAHs intake in the dust samples varied as children summer: 16.2 ng kg^−1^ day^−1^, winter: 16.3 ng kg^−1^ day^−1^, adults summer: 0.01 ng kg^−1^ day^−1^, winter: 2.5 ng kg^−1^ day^−1^ (Fig. 5a). Total EDI range of breathing intake of air and dust PAHs for two age groups ranged from 695.1 to 1362.7 ng kg^−1^ day^−1^ during the study period. EDI for breathing intake of PAHs in air samples varied as children summer: 1317 ng kg^−1^ day^−1^, winter: 1362.7 ng kg^−1^ day^−1^ and adults summer: 695.1 ng kg^−1^ day^−1^, winter: 719.2 ng kg^−1^ day^−1^. Whereas, total EDI in dust samples were found to be children: 1234.5 ng kg^−1^ day^−1^, 1235.8 ng kg^−1^ day^−1^ and adults: 260 ng kg^−1^ day^−1^, 652.2 ng kg^−1^ day^−1^ during summer and winter seasons, respectively (Fig. 5b). The pattern for PAHs intake values through both oral and breathing exposure was higher in children than adults because PAHs can enter the body by breathing in the air contaminated with dust, cigarette smoke, wood, coal, or any other anthropogenic activity such as mining, oil refining, metallurgy, chemical manufacture, transportation, and the electrical sector. PAHs are inhaled through the lungs and are largely deposited in the kidneys, liver and fat. The present study's findings were consistent with previous research conducted in China and Pakistan^10, 94^.

## Practical implications of this study

As a developing country, Pakistan is facing serious energy crises, and to generate required energy has adopted alternate inefficient fuels, which results in environmental deterioration, which substantially increases the PAHs emissions. Furthermore, the paradigm shift of natural gas vehicles to gasoline/diesel fuel engines has also substantially contributed to the outdoor concentration of PAHs^10^. Additionally, due to a shortage of natural gas (comparatively cleaner fuel), biomass combustion became the main source of household required energy, resulting in an upsurge of pyrogenic PAHs^41, 72^. As few past studies highlighted the significance of long-term strategies that intend to transition from allocating subsidies to unsustainable, environmentally-degrading fossil fuels to sustainably-produced renewable energy carriers^10, 42, 95, 96^. Therefore, The green technologies and dissemination of alternative fuels mainly biodiesel and solar power could be one of the environment-friendly alternatives and planning infrastructure, fuel quality, fuel subsidies, renewable energy industry, energy price change and abatement of industrial emissions will be highly essential to reduce the PAHs pollution and maintain the air quality to boost up the economy and achieve energy security of the city.

## Conclusions and recommendations

The present study assessed sixteen US EPA priority listed PAHs in outdoor air and dust environments from ten selected areas of Lahore city, Pakistan. Results derived from the comparative analysis identified relative higher PAHs concentrations during the winter season in both air and dust matrices, with air being a more contaminated environmental compartment, which can be attributed to diesel combustion and heavy traffic. Naphthalene, Phenanthrene and Pyrene were the primary PAHs contributors to the air and dust PAHs in Lahore City. According to the particular isomer ratios, PAHs in the investigated region were largely produced by fuel combustion as well as petroleum emissions. Ingestion and dermal contact were the primary exposure routes for PAHs long term exposure. In comparison, inhalation was the least significant contributor to air and dust matrices. The estimated total ILCR from Σ16PAHs exposure signifies a high health risk to the exposed population. This research identifies the need for immediate actions of legislation to limit the semi-volatile organic compounds, particularly PAHs, in urban cities of the developing world and enhance environmental management and health conditions.

Overpopulation, rapid industrialization and urbanization have challenged the energy resources and resulted in a dramatic shift of non-environment friendly fuel choice, which has elevated the PAHs levels in the city. Therefore, the investigation of the gaseous PAHs in the atmosphere and dust of the second largest city of Pakistan suggests that green technologies should be introduced in the market to reduce the gap between energy need and supply and ensure public health. Furthermore, the government should formulate policies to minimize pollution load and improve air quality and associated health risks. Moreover, comprehensive research, including a wide range of environmental matrices with varying socioeconomic factors, is needed to determine the extent of chemical contamination in the world's worst air quality affected city, i.e. Lahore.

## Supplementary Information

Supplementary Information.

## Data Availability

All data generated or analyzed during this study are included in this article (and its supplementary information file).

## Abbreviations

PAHs: Polycyclic aromatic hydrocarbons
GS/MS: Gas chromatography/mass spectrometry
ILCR: Incremental lifetime cancer risk
HMW: High molecular weight
LMW: Low molecular weight
US EPA: United States Environmental Protection Agency
WHO: World Health Organization
PAS: Passive air samplers
EPD: Environment Protection Department
PUF: Polyurethane foam
DCM: Dichloromethane
AASHTO: American Association of State Highway and Transportation Officials
SIM: Split injection mode
TEF: Toxic equivalency factor
BaP-TEQ: Benzo(a)pyrene toxicity equivalent
BW: Body weight
CSF: Carcinogenic slope factor
EF: Exposure frequency
ED: Exposure duration
EDI: Estimated daily intake
