# Supplementary material for: Source identification and risk assessment of polycyclic aromatic hydrocarbons (PAHs) in air and dust samples of Lahore City
Source: Sci Rep. 2022 Feb 14;12:2459. doi: 10.1038/s41598-022-06437-8 (PMC8844380; doi:10.1038/s41598-022-06437-8)
Supplement: Supplementary file 1 — Supplementary Information. [file 41598_2022_6437_MOESM1_ESM.docx]

**Text S1. Calculation method for PUF standard passive air sampling rate**

**PUF-PAS rate calculations**

The amount of PAHs (M_PUF_, ng) deposited on PUF media is determined by a mass transfer coefficient and a concentration gradient between the air and the PUF disc.

dM_PUF_dt = kivas⋅(C_Air_−C_PUF_ K_PUF_) Eq. 1

C*_Air_* = M_PUF_(k_v_A_s_t)^−1^ Eq. 2

*where A_s_* = PUF surface area (m^2^), *k_v_* = mass transfer coefficient (m/s), *C_Air_* = analyte concentration in the air (ng/m^3^), *C_PUF_* = PAHs concentration on the PUF (ng/m^3^) and K_PUF_ = dimensionless PUF/air equilibrium partition coefficient.

Because K_PUF_ for PAHs is enormous, the mass of PAHs on the PUF is a linear function of time. The PAHs concentrations in air measured by the passive sampler can be calculated using Equation 2, considering that C*_Air_* is a constant.

The product *k_v_*·*A_s_* must be determined by experiment and is called the sampling rate, R-value.

The R-value derived from depuration compound loss is computed by calculating the mass transfer coefficient as a function of the first-order elimination rate constant (k_e_, sec^−1^) and the stagnant air layer over the PUF surface (*δ_film_,* m) as specified by Shoeib and Harner (2002):

k_v_ = k_e_ · K_PUF_ · δ_film_ Eq. 3

Where

ke=ln(m/m0)/t Eq. 4

R-values may be calculated from the linear uptake curve of native congeners from the ambient air of known concentration:

R=b/C_Air_ Eq. 5

Here b is the slope of mass accumulated versus time^1^.


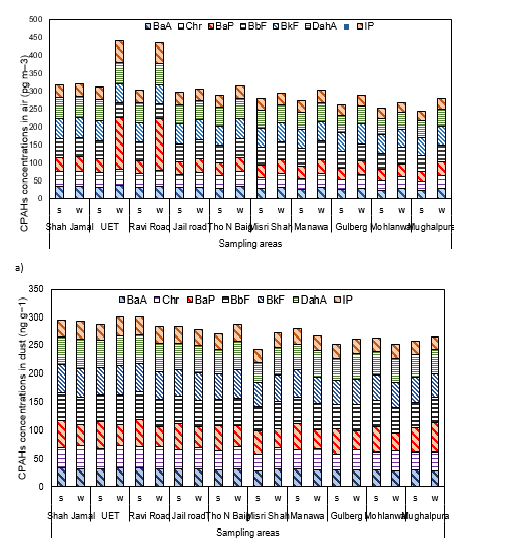


**Figure S1: Mean carcinogenic ∑C_7_PAHs concentrations in (a) air (pg m^–3^) and (b) in dust (ng g^−1^) during summer and winter seasons in Lahore city**

**Table S1: Parameters used for estimation of Incremental Life Cancer risk from urban air and dust bound PAHs**

| **Exposure Parameter** | **Abbreviation** | **Values** | **Unit** | **Reference** |
| --- | --- | --- | --- | --- |
| Ingestion rate | Ing R | Child= 200; Adult= 100 | mg day^-1^ | US EPA 2009^2^ |
| Exposure frequency | EF | 350 | days year^-1^ | ---------------- |
| Exposure duration | ED | Child= 6; Adult= 30 | years | US EPA 1999^3^ |
| Body Weight | BW | Child= male:14.3; female:13.6  Adult= male:62.8; female:54.7 | kg | ……………… |
| Average lifetime | AT (h)  AT (d) | 70 years × 365 dayy^-1^ × 24hr = 613200  70×365 dayy^-1^=25550 |  | US EPA 2009^2^ |
| Carcinogenic slope factor | CSF | CSF ingestion =7.3  CSF dermal = 25  CSF inhalation = 3.85 | (mg kg^−1^ day^−1^)^−1^ | Kamal et al. (2015)^4^ |
| Particle emission factor | PEF | 1.36×10^9^ | m^3^kg^-1^ | US EPA 2013^4^ |
| Inhalation Rate | InhR | 20 | m^3^ day^-1^ | Kamal et al. (2014^5^, 2015^4^) |
| Exposed skin area | SA | Child= 2800; Adult= 5700 | cm^2^ | US EPA 2009^2^ |
| dermal adherence factor | AF | Child= 0.2; Adult= 0.007 | mg/cm^-2^ | US EPA 2009^2^ |
| Dermal adsorption factor | ABS | 0.13 | unit less | US EPA 2009^1^ & Kamal et al. (2015)^3^ |

**Table S2: Summer (min-max) PAHs concentrations in Air (pg m^–3^)**

| **16**  **PAHs**  **Abbr** | **Sampling areas** | | | | | | | | | | | |
| --- | --- | --- | --- | --- | --- | --- | --- | --- | --- | --- | --- | --- |
|  | **Rings** | **Shah Jamal** | **UET** | **Ravi Road** | **Jail road** | **Tho N Baig** | **Misri Shah** | **Manawa** | **Gulberg** | **Mohlanwal** | **Mughalpura** |  |
| Naph | 2 | 100.4-186.4 | 99.8-185.3 | 99.1-184.1 | 98.8-183.6 | 98.6-183.0 | 98.2-182.4 | 97.9-181.7 | 96.7-179.7 | 96.2-178.6 | 95.6-177.5 |  |
| Ace | 3 | 19.3-35.9 | 18.8-35.0 | 18.3-34.1 | 18.0-33.4 | 17.7-32.9 | 17.4-32.2 | 17.0-31.6 | 16.5-30.6 | 15.9-29.5 | 15.3-28.3 |  |
| Acy | 3 | 8.3-15.3 | 7.9-14.7 | 7.6-14.2 | 7.4-13.7 | 6.9-12.9 | 6.6-12.2 | 6.4-11.8 | 6.1-11.3 | 6.0-11.1 | 5.7-10.5 |  |
| Ant | 3 | 16.4-30.4 | 15.6-29.0 | 15.3-28.5 | 14.6-27.2 | 13.8-25.6 | 13.0-24.1 | 12.3-22.8 | 11.4-21.2 | 9.9-18.5 | 9.7-18.1 |  |
| Flu | 3 | 26.4-49.0 | 26.3-48.9 | 26.0-48.4 | 25.6-47.6 | 25.4-47.2 | 25.3-47.1 | 24.9-46.2 | 24.4-45.4 | 24.0-44.6 | 23.4-43.4 |  |
| Phe | 3 | 114.6-212.8 | 113.9-211.5 | 113.3-210.5 | 112.6-209.2 | 111.6-207.2 | 111.0-206.2 | 109.5-203.3 | 108.2-200.9 | 107.7-200.1 | 106.8-198.4 |  |
| BaA | 4 | 23.4-43.4 | 22.8-42.3 | 21.9-40.7 | 21.5-39.9 | 20.8-38.6 | 19.2-35.6 | 18.8-34.8 | 17.2-31.9 | 15.8-29.4 | 14.9-27.7 |  |
| Chr | 4 | 29.3-54.5 | 27.9-51.7 | 26.0-48.2 | 24.9-46.2 | 23.5-43.7 | 21.9-40.7 | 20.7-38.5 | 19.5-36.3 | 17.8-33.0 | 16.7-30.9 |  |
| Fla | 4 | 100.3-186.3 | 100.0-185.8 | 98.4-182.7 | 97.2-180.4 | 96.3-178.9 | 95.8-178.0 | 95.1-176.7 | 94.1-174.7 | 93.0-172.6 | 91.2-169.4 |  |
| Pyr | 4 | 78.7-146.1 | 77.4-143.7 | 76.0-141.2 | 74.8-138.8 | 73.4-136.2 | 71.9-133.5 | 70.4-130.7 | 69.2-128.6 | 67.8-125.8 | 66.7-123.9 |  |
| BaP | 5 | 26.8-49.8 | 26.3-48.9 | 25.8-48.0 | 25.6-47.6 | 25.3-47.1 | 25.0-46.4 | 24.4-45.4 | 23.5-43.7 | 23.0-42.8 | 22.2-41.2 |  |
| BbF | 5 | 37.0-68.6 | 36.7-68.1 | 36.3-67.3 | 35.5-65.9 | 34.9-64.7 | 34.2-63.6 | 33.5-62.3 | 32.5-60.3 | 31.8-59.0 | 30.7-56.9 |  |
| BkF | 5 | 39.1-72.7 | 39.0-72.4 | 38.4-71.2 | 38.2-71.0 | 37.7-70.1 | 37.5-69.7 | 37.0-68.8 | 36.8-68.3 | 36.0-66.8 | 35.3-65.5 |  |
| DahA | 5 | 41.4-77.0 | 40.9-75.9 | 39.7-73.7 | 38.2-71.0 | 36.8-68.4 | 35.6-66.0 | 34.8-64.6 | 33.3-61.9 | 32.1-59.7 | 31.7-58.9 |  |
| IP | 6 | 25.3-47.1 | 25.1-46.7 | 24.4-45.2 | 23.5-43.7 | 22.8-42.3 | 22.3-41.5 | 21.5-39.9 | 20.9-38.9 | 19.8-36.8 | 19.2-35.6 |  |
| BghiP | 6 | 20.9-38.9 | 20.4-38.0 | 19.9-36.9 | 19.7-36.7 | 19.3-35.8 | 19.0-35.2 | 18.6-34.6 | 18.4-34.2 | 17.8-33.0 | 17.6-32.6 |  |

**Table S3: Winter (min-max) PAHs concentrations in Air (pg m^–3^)**

| **16 PAHs**  **Abbr** | **Sampling areas** | | | | | | | | | | |
| --- | --- | --- | --- | --- | --- | --- | --- | --- | --- | --- | --- |
|  | **Rings** | **Shah Jamal** | **UET** | **Ravi Road** | **Jail road** | **Tho N Baig** | **Misri Shah** | **Manawa** | **Gulberg** | **Mohlanwal** | **Mughalpura** |
| Naph | 2 | 101.2-187.9 | 102.1-189.5 | 101.9-189.2 | 100.2-186.2 | 100.9-187.5 | 99.1-184.0 | 99.6-185.0 | 98.4-182.7 | 97.0-180.1 | 97.6-181.2 |
| Ace | 3 | 19.8-36.8 | 20.4-37.8 | 20.2-37.4 | 19.2-35.6 | 19.5-36.3 | 17.9-33.3 | 18.6-34.5 | 17.2-31.9 | 16.2-30.0 | 16.5-30.6 |
| Acy | 3 | 8.3-15.3 | 8.9-16.5 | 8.5-15.9 | 7.4-13.8 | 7.9-14.7 | 6.7-12.5 | 7.3-13.5 | 6.0-11.2 | 5.0-9.4 | 5.5-10.1 |
| Ant | 3 | 17.2-32.0 | 18.5-34.3 | 17.9-33.2 | 15.5-28.9 | 16.4-30.4 | 14.2-26.4 | 14.9-27.7 | 13.6-25.2 | 11.6-21.6 | 13.1-24.3 |
| Flu | 3 | 27.2-50.6 | 27.9-51.7 | 27.5-51.1 | 26.2-48.6 | 26.9-49.9 | 25.1-46.7 | 25.6-47.6 | 24.9-46.3 | 24.0-44.6 | 24.4-45.4 |
| Phe | 3 | 114.2-212.2 | 116.1-215.5 | 115.2-213.9 | 113.1-210.0 | 113.7-211.1 | 111.7-207.5 | 112.4-208.7 | 111.0-206.1 | 109.5-203.3 | 110.1-204.5 |
| BaA | 4 | 24.1-44.7 | 25.6-47.6 | 24.7-45.9 | 22.7-42.1 | 23.6-43.8 | 21.5-39.9 | 22.1-41.0 | 20.5-38.1 | 19.4-36.0 | 20.0-37.2 |
| Chr | 4 | 29.5-54.9 | 30.7-57.1 | 30.5-56.7 | 28.2-52.4 | 29.3-54.3 | 27.2-50.6 | 27.5-51.1 | 26.4-49.0 | 24.1-44.7 | 25.6-47.6 |
| Fla | 4 | 100.2-186.2 | 101.6-188.6 | 101.2-187.9 | 98.8-183.4 | 99.6-185.0 | 97.7-181.4 | 98.3-182.5 | 96.9-179.9 | 95.3-177.1 | 96.3-178.8 |
| Pyr | 4 | 73.1-135.7 | 74.3-137.9 | 73.7-136.9 | 71.8-133.3 | 72.2-134.2 | 70.6-131.0 | 70.8-131.6 | 69.7-129.4 | 68.2-126.6 | 69.0-128.2 |
| BaP | 5 | 27.8-51.6 | 28.5-52.9 | 28.1-52.3 | 27.2-50.6 | 27.5-51.1 | 26.4-49.0 | 26.9-49.9 | 26.2-48.6 | 24.1-44.7 | 24.9-46.3 |
| BbF | 5 | 37.2-69.0 | 38.0-70.6 | 37.5-69.6 | 36.5-67.7 | 36.7-68.1 | 35.3-65.5 | 36.1-67.0 | 34.5-64.1 | 32.5-60.3 | 34.2-63.4 |
| BkF | 5 | 39.7-73.7 | 40.5-75.3 | 40.2-74.6 | 38.8-72.0 | 39.3-73.1 | 38.2-70.9 | 38.4-71.4 | 37.6-69.8 | 35.9-66.7 | 36.6-68.0 |
| DahA | 5 | 40.3-74.8 | 42.9-79.7 | 41.5-77.1 | 37.4-69.4 | 38.7-71.9 | 35.1-65.3 | 37.0-68.6 | 34.9-64.7 | 31.9-59.2 | 33.3-61.9 |
| IP | 6 | 25.6-47.5 | 27.2-50.4 | 26.2-48.6 | 24.0-44.6 | 24.9-46.2 | 22.8-42.3 | 23.5-43.6 | 22.0-40.8 | 19.2-35.6 | 20.5-38.1 |
| BghiP | 6 | 20.7-38.5 | 22.1-41.0 | 21.8-40.4 | 19.8-36.8 | 20.5-38.1 | 19.5-36.1 | 19.7-36.5 | 19.3-35.8 | 18.6-34.5 | 18.8-34.8 |

**Table S4: Summer (min-max) PAHs concentrations in Dust (ng g^−1^)**

| 16PAHs  Abbr | Sampling areas | | | | | | | | | | |
| --- | --- | --- | --- | --- | --- | --- | --- | --- | --- | --- | --- |
|  | Rings | Shah Jamal | UET | Ravi Road | Jail road | Tho N Baig | Misri Shah | Manawa | Gulberg | Mohlanwal | Mughalpura |
| Naph | 2 | 93.2-173.2 | 92.9-172.5 | 94.0-174.6 | 92.4-171.6 | 90.9-168.7 | 88.8-164.8 | 91.6-170.2 | 88.9-165.1 | 90.1-167.3 | 89.3-165.9 |
| Ace | 3 | 16.0-29.8 | 15.1-28.0 | 16.2-30.2 | 14.8-27.4 | 13.4-25.0 | 12.6-23.4 | 13.8-25.6 | 12.7-23.7 | 13.3-24.7 | 13.0-24.1 |
| Acy | 3 | 6.0-11.2 | 5.7-10.5 | 6.2-11.6 | 5.3-9.8 | 5.0-9.2 | 4.2-7.8 | 5.1-9.5 | 4.4-8.2 | 4.8-9.0 | 4.6-8.5 |
| Ant | 3 | 15.6-29.0 | 15.3-28.5 | 16.0-29.6 | 15.0-27.8 | 14.6-27.0 | 11.3-21.1 | 14.7-27.3 | 12.0-22.4 | 13.7-25.4 | 12.8-23.8 |
| Flu | 3 | 23.7-43.9 | 24.4-45.4 | 24.0-44.6 | 23.5-43.7 | 21.8-40.6 | 20.5-38.1 | 22.6-42.0 | 21.6-40.2 | 21.4-39.7 | 20.7-38.4 |
| Phe | 3 | 113.7-211.1 | 113.3-210.5 | 114.7-212.9 | 112.6-209.2 | 110.3-204.9 | 106.8-198.3 | 111.6-207.2 | 107.7-200.1 | 109.6-203.5 | 108.8-202.0 |
| BaA | 4 | 23.5-43.6 | 22.8-42.4 | 23.7-44.1 | 23.0-42.8 | 22.0-40.8 | 20.5-38.1 | 22.9-42.5 | 20.8-38.6 | 21.6-40.0 | 21.4-39.7 |
| Chr | 4 | 25.8-47.8 | 24.6-45.6 | 26.1-48.5 | 24.2-44.9 | 22.6-42.0 | 20.3-37.7 | 23.5-43.7 | 20.6-38.2 | 22.3-41.5 | 21.6-40.2 |
| Fla | 4 | 96.3-178.9 | 95.6-177.5 | 97.0-180.1 | 95.1-176.5 | 91.6-170.2 | 86.0-159.6 | 93.5-173.7 | 87.4-162.2 | 90.2-167.6 | 88.5-164.3 |
| Pyr | 4 | 65.5-121.6 | 64.8-120.4 | 66.4-123.4 | 64.3-119.3 | 62.8-116.6 | 59.7-110.9 | 63.6-118.0 | 60.8-112.8 | 62.0-115.1 | 61.5-114.3 |
| BaP | 5 | 34.0-63.2 | 33.5-62.3 | 34.2-63.6 | 33.3-61.9 | 32.0-59.4 | 29.5-54.7 | 32.8-61.0 | 30.7-57.1 | 31.6-58.6 | 31.2-58.0 |
| BbF | 5 | 33.9-62.9 | 33.5-62.1 | 34.2-63.6 | 33.1-61.5 | 32.1-59.7 | 29.6-55.0 | 32.8-60.8 | 30.5-56.6 | 31.6-58.8 | 31.1-57.7 |
| BkF | 5 | 34.1-63.3 | 33.7-62.5 | 34.4-64.0 | 33.3-61.9 | 31.9-59.3 | 29.3-54.3 | 32.8-61.0 | 29.7-55.1 | 31.4-58.4 | 30.5-56.6 |
| DahA | 5 | 34.6-64.2 | 33.4-62.0 | 35.6-66.0 | 32.6-60.6 | 30.0-55.6 | 25.0-46.4 | 31.2-58.0 | 27.2-50.6 | 28.5-52.9 | 27.7-51.5 |
| IP | 6 | 21.1-39.3 | 20.4-38.0 | 22.3-41.3 | 20.2-37.4 | 18.6-34.6 | 15.6-29.0 | 19.5-36.1 | 16.5-30.7 | 17.8-33.0 | 16.9-31.3 |
| BghiP | 6 | 16.5-30.6 | 15.8-29.4 | 16.6-30.8 | 15.5-28.7 | 15.2-28.2 | 13.7-25.4 | 15.3-28.5 | 14.1-26.1 | 14.9-27.7 | 14.4-26.7 |

**Table S5: Winter (min-max) PAHs concentrations in Dust (ng g^−1^)**

| 16PAHs  Abbr | Sampling areas | | | | | | | | | | |
| --- | --- | --- | --- | --- | --- | --- | --- | --- | --- | --- | --- |
|  | Rings | Shah Jamal | UET | Ravi Road | Jail road | Tho N Baig | Misri Shah | Manawa | Gulberg | Mohlanwal | Mughalpura |
| Naph | 2 | 96.9-179.9 | 97.5-181.1 | 96.9-179.9 | 96.0-178.4 | 97.5-181.1 | 95.7-177.7 | 95.1-176.5 | 94.0-174.6 | 93.0-172.8 | 93.2-173.0 |
| Ace | 3 | 17.6-32.6 | 17.9-33.2 | 17.2-32.0 | 16.9-31.5 | 17.4-32.4 | 16.4-30.4 | 16.2-30.0 | 15.9-29.5 | 14.8-27.4 | 15.3-28.3 |
| Acy | 3 | 6.5-12.1 | 6.7-12.4 | 6.2-11.4 | 6.0-11.1 | 6.4-11.8 | 5.7-10.5 | 5.3-9.9 | 5.1-9.5 | 4.6-8.5 | 5.0-9.4 |
| Ant | 3 | 16.3-30.3 | 16.7-30.9 | 14.6-27.2 | 14.4-26.7 | 14.8-27.6 | 13.9-25.9 | 13.0-24.2 | 12.4-23.0 | 11.1-20.5 | 11.8-21.8 |
| Flu | 3 | 25.4-47.2 | 25.8-47.8 | 24.9-46.2 | 24.6-45.6 | 25.1-46.7 | 24.4-45.2 | 23.9-44.3 | 23.7-43.9 | 23.2-43.0 | 23.5-43.6 |
| Phe | 3 | 112.8-209.6 | 113.9-211.5 | 111.4-207.0 | 111.0-206.2 | 112.3-208.5 | 110.3-204.8 | 109.7-203.7 | 109.1-202.5 | 107.7-199.9 | 108.4-201.2 |
| BaA | 4 | 23.3-43.3 | 23.7-43.9 | 23.0-42.8 | 22.8-42.4 | 23.2-43.0 | 22.7-42.1 | 22.1-41.0 | 21.9-40.7 | 20.5-38.1 | 20.7-38.5 |
| Chr | 4 | 27.5-51.1 | 27.9-51.9 | 26.7-49.5 | 26.1-48.5 | 26.9-49.9 | 25.5-47.3 | 25.0-46.4 | 24.4-45.2 | 23.5-43.6 | 23.9-44.5 |
| Fla | 4 | 97.7-181.4 | 99.2-184.2 | 95.0-176.4 | 93.5-173.6 | 96.1-178.5 | 91.9-170.7 | 90.7-168.5 | 89.4-166.0 | 87.4-162.2 | 88.1-163.7 |
| Pyr | 4 | 67.4-125.2 | 68.4-127.0 | 66.1-122.7 | 65.5-121.7 | 66.7-123.9 | 64.8-120.3 | 64.3-119.3 | 63.6-118.2 | 62.1-115.3 | 62.5-116.1 |
| BaP | 5 | 26.3-48.8 | 26.5-49.3 | 25.6-47.6 | 25.4-47.2 | 26.1-48.5 | 24.6-45.6 | 24.3-45.1 | 23.5-43.7 | 36.3-67.5 | 23.0-42.6 |
| BbF | 5 | 34.6-64.2 | 35.6-66.0 | 34.0-63.1 | 33.3-61.8 | 34.2-63.6 | 33.2-61.6 | 32.8-60.8 | 32.1-59.5 | 30.7-57.1 | 31.2-57.9 |
| BkF | 5 | 35.2-65.4 | 36.2-67.2 | 34.0-63.2 | 33.4-62.0 | 34.6-64.2 | 32.8-60.8 | 31.9-59.3 | 31.4-58.2 | 29.3-54.3 | 30.7-56.9 |
| DahA | 5 | 35.8-66.6 | 36.7-68.1 | 34.7-64.5 | 34.0-63.1 | 35.2-65.4 | 33.4-62.0 | 32.7-60.7 | 32.1-59.7 | 30.0-55.8 | 30.5-56.7 |
| IP | 6 | 22.7-42.1 | 23.4-43.4 | 20.7-38.5 | 20.0-37.1 | 21.3-39.5 | 19.5-36.1 | 18.8-34.8 | 18.1-33.7 | 15.2-28.2 | 16.7-30.9 |
| BghiP | 6 | 18.4-34.2 | 18.6-34.5 | 17.6-32.8 | 17.4-32.2 | 18.0-33.4 | 17.0-31.6 | 16.5-30.6 | 16.0-29.8 | 14.4-26.7 | 15.0-27.8 |

**Table S6: Comparative table of ∑PAHs concentrations and methods in air (pg m^-3^) reported worldwide**

| Locations | Number of PAHs | PAHs concentrations in air (pg m^-3^) Mean (Min-Max) | Methodology/Instrumentation | References |
| --- | --- | --- | --- | --- |
| Chennai, India | 11 | 517100 (121100–1370500) | Fine Particulate Sampler with PTFE membrane filters was used for air sampling from March 2009 to February 2010 at monthly intervals. | (Mohanraj et al. 2010)^6^ |
| Tiruchirappalli, India | 9 | 259400(136000–487500) | Fine Particulate Sampler with PTFE membrane filters was used for air sampling from March 2009 to February 2010 at monthly intervals. | (Mohanraj et al. 2011)^7^ |
| Ningbo, China | 16 | 46000(11000-103000) | Particulate phase PAHs were captured on PALL quartz [microfiber](about:blank) filters and gaseous PAHs were adsorbed [polyurethane foam](about:blank). Samples were collected once every week from July 2009 to March 2010 on the roof of a building. | (Liu et al. 2014)^8^ |
| Silesia, Poland | 15 | (22800-107800) | Quartz fiber filters (QMA, 47 mm in diameters) were used to collect outdoor and indoor air samples during the spring season (17 March to 3 May 2010). | (Błaszczyk et al. 2017)^9^ |
| Tianjin, China | 16 | (27300-58200) | Personal monitoring was performed throughout one year using a backpack with PEM (PEM-PM2.5; BGI inc., Waltham, MA) inlets, equipped with one pump connected to one sampler, loaded with a 37 mm quartz filter. | (Han et al. 2014)^10^ |
| Khatmandu, Nepal | 15 | 155000(18100-453000) | TSP filter sampler was placed on the rooftop (15 m above ground) of a residential building. Fifty TSP samples were collected on pre-baked quartz fiber filters by using a filter sampler fitted with a TSP cyclone Each sample was collected for 24 h every 8 days in a year to check seasonal variation. | (Chen et al. 2015)^11^ |
| Serbia, Europe | 16 | (27000-132000) | Polyurethane foam (PUF) was used to collect gas phase of PAHs. Outdoor and indoor samples were collected during December 2011 and Jun 2012. | (Živković et al. 2015)^12^ |
| Tehran, Iran | 16 | 57000 (56980-171250) | The SKC sampling pump was used equipped with a PTFE filter. The duration of sampling was a consecutive 24 h on a biweekly base and a total of 112 samples were collected during the summer 2013 and winter 2014. | (Hoseini et al. 2016)^13^ |
| Paris, France | 16 | 1000 (10000-100000) | The particulate phases were sampled onto quartz fibre filters and gaseous phases onto XAD-2 resin (20–60 mesh).sampling was carried out from summer 2010 till winter 2012. | (Teil et al. 2016)^14^ |
| Lampang, Thailand | 15 | (4200-224000) | Personal air sampler with an ATPS-20H impactor connected to a portable MP-Σ300 pump was used for three times over 24 h sampling, conducted for 3 days between 9th and 12th of March in 2013. | (Orakij et al. 2017)^15^ |
| Pindi&Islamabad, Pakistan | 16 | 2132 (1630–2893) | 25 [Polyurethane foam](about:blank) (PUF) passive air samplers (PUF-PAS) were deployed at 8 sampling sites for outdoor and indoor air sampling for 56 days from 15th February to 22nd April 2014. | (Hamid et al. 2018)^16^ |
| Hong Kong, China | 26 | 1700 (400-5200) | 60 outdoor and 64 indoor air samples were collected during 65 days using two Mini-Volume air samplers side-by-side, loaded with one Teflon and one quartz-fiber filter. | (Chen et al. 2020)^17^ |
| Xian, China | 19 | 116000 (32000-224000) | 24 h indoor and personal exposure (PE) samples were collected for three consecutive days in each household from January 11 to 26, 2018 | (He et al. 2021)^18^ |
| Lahore, Pakistan | 16 | 956.6 (860.7-1035.8) | 10 PUF-PAS passive air samplers were deployed at 10 sampling sites for consecutive 56-days during two sampling periods in year 2017 and 2018. | This study |

**Table S7: Comparative table of ∑PAHs concentrations and methods in dust (ng g^-1^) reported worldwide**

| **Locations** | **Number of PAHs** | **PAHs concentrations in dust (ng g^-1^) Mean (Min-Max)** | **Methodology/Instrumentation** | **References** |
| --- | --- | --- | --- | --- |
| Lanzhou, China | 16 | 3900 (1240-10,700) | 32 street dust samples were collected using [polyethylene](about:blank) brush, tray and containers in October 2011. | (Jiang et al. 2014)^19^ |
| Sydney, Australia | 16 | 2910(1650-4000) | 29 dust samples were collected from July 2012 to January 2013. | (Nguyen et al. 2014)^20^ |
| Chung, Punjab, Pakistan | 16 | 882 (692–1007) | 20 g of the dust particles collected using plastic brushes and dustpans from 26 brick kiln units during year 2014. | (Kamal et al. 2014)^5^ |
| Ulsan, Korea | 16 | 960 (65-12,000) | 5 sub-soil samples of each 100 g were collected from 25 industrial, urban and rural sites in July 2010. | (Kwon and Choi 2014)^21^ |
| New Delhi, India | 23 | 1100 (650–1700) | 300 g of 7 Street dust samples were collected by using broom during 2012–2013. | (Tue et al. 2014)^22^ |
| Tianjin, China | 16 | 7993.3(538–34,300) | 5 to 7 subsamples (each 200g) were collected on pavements next to roads by using brushes and trays at 87 sampling sites in May 2011. | (Yu et al. 2014)^23^ |
| Isfahan, Iran | 16 | 1074.58(184.6-3221.7) | 24 road dust samples (each 500 g) were collected from airport, refinery, ringway, terminal and highway police station during August 2012 by using a plastic hand broom and transferred to a clean and sealed polyethylene bag. | (Soltani et al. 2015)^24^ |
| Xi'an, China | 29 | 15767(10,422–26,258) | 20 urban road dusts and 6 suburban surface soil samples were collected in 2015. | (Wei et al. 2015)^25^ |
| Jeddah, SaudiArabia | 12 | 1674 (950–11,950) | Indoor dust samples from household floor, AC filter and vehicle dust (15 each) were collected during 2014–2015. For household floor dust vacuum cleaner bags were used and AC filters were cleaned with brush on aluminum foil to collect dust samples. | (Ali et al. 2016)^26^ |
| Kumasi, Ghana | 28 | 2570(181–7770) | 20 g of each Street dust samples were collected using a brush and a pan. | (Bandowe et al. 2016)^27^ |
| Pindi &Islamabad, Pakistan | 16 | 89.8(37.1-244) | 10 g of each outdoor and indoor dust samples (comprised of 5 individual subsamples) collected from sampling locations by using plastic brushes and dustpans. | (Hamid et al. 2018)^16^ |
| Bandar Abbas, Iran | 16 | 362300(73080-1339000) | 27 dust samples (each 500 g) were collected from street surfaces of sampling sites during January 2016. Sampling involved gently sweeping by plastic hand and self-sealed in polyethylene bag. | (Keshavarzi et al. 2018)^28^ |
| Mashhad, Iran | 16 | 2183.5(764-8986.7) | 22 road dust samples (each of 500 g) were collected using [polyethylene](about:blank) brushes from the urban areas from July to August 2014. Collected samples were transferred to a sealed polyethylene bag before transport to the laboratory. | (Najmeddin et al. 2018)^29^ |
| Shanghai, China | 16 | 1552(223-8214) | The agricultural soil samples were collected from 26 sampling locations within 5 km of plants in the suburbs in March 2016. | (Tong et al. 2018)^30^ |
| Huanggang, China | 16 | 1862.1(622.9-4340.7) | 100 g of each 21 street dust samples were collected within a 5 m radius circle by plastic brushes and dustpans in October 2013. The sampling locations were classified as traffic area, business district, residential and education area. | (Liu et al. 2019)^31^ |
| Karaj, Iran | 16 | 624(16.2-1236.2) | 30 street dust samples (each 250 g) were collected from different functional areas during the winter of 2017 (from 15th December to 15th November) by using polyethylene brush and swept into a dustpan. | (Qishlaqi and Beiramali 2019)^32^ |
| Lahore, Pakistan | 16 | 895(818.9-963.4) | 5g of dust samples from 10 sampling locations were collected during winter (October-March) and summer (April-September) in year 2017 and 2018. Samples were gathered in stainless steel dustpans by using plastic brushes in a gentle sweeping movement. | This study |

**References**

1. Shoeib, M., Harner, T. Characterization and comparison of three passive air samplers for persistent organic pollutants. *Environmental science & technology*. **36** (19), 4142-4151 (2002).
2. Bishoi, B., Prakash, A. & Jain, V. KA comparative study of air quality index based on factor analysis and US-EPA methods for an urban environment. *Aerosol and Air Quality Research*. **9** (1), 1-17 (2009).
3. Doull, J., Cattley, R., Elcombe, C., Lake, B. G., Swenberg, J., Wilkinson, C. ... & Van Gemert, M. (1999). A cancer risk assessment of di (2-ethylhexyl) phthalate: application of the new US EPA Risk Assessment Guidelines. *Regulatory Toxicology and Pharmacology*. **29** (3), 327-357 (1999).
4. Kamal, A., Malik, R. N., Martellini, T. & Cincinelli, A. Source, profile, and carcinogenic risk assessment for cohorts occupationally exposed to dust-bound PAHs in Lahore and Rawalpindi cities (Punjab province, Pakistan). *Environmental Science and Pollution Research*. **22** (14), 10580-10591 (2015).
5. Kamal, A., Malik, R. N., Martellini, T. & Cincinelli, A. Cancer risk evaluation of brick kiln workers exposed to dust bound PAHs in Punjab province (Pakistan). *Science of the total environment*. ***493***, 562-570 (2014).
6. Mohanraj, R., Solaraj, G. & Dhanakumar, S. PM 2.5 and PAH concentrations in urban atmosphere of Tiruchirappalli, India. *Bulletin of Environmental Contamination and Toxicology*. **87** (3), 330-335 (2011).
7. Mohanraj, R., Solaraj, G. Dhanakumar, S. Fine particulate phase PAHs in ambient atmosphere of Chennai metropolitan city, India. *Environmental Science and Pollution Research*. **18** (5), 764-771 (2011).
8. Liu, D., Xu, Y., Chaemfa, C., Tian, C., Li, J., Luo, C. & Zhang, G. Concentrations, seasonal variations, and outflow of atmospheric polycyclic aromatic hydrocarbons (PAHs) at Ningbo site, Eastern China. *Atmospheric Pollution Research*. **5** (2), 203-209 (2014).
9. Błaszczyk, E., Rogula-Kozłowska, W., Klejnowski, K., Fulara, I. & Mielżyńska-Švach, D. Polycyclic aromatic hydrocarbons bound to outdoor and indoor airborne particles (PM2. 5) and their mutagenicity and carcinogenicity in Silesian kindergartens, Poland. *Air Quality, Atmosphere & Health*. **10** (3), 389-400 (2017).
10. Han, J., Zhang, N., Niu, C., Han, B. & Bai, Z. Personal exposure of children to particle-associated polycyclic aromatic hydrocarbons in Tianjin, China. *Polycyclic Aromatic Compounds*.**34** (4), 320-342 (2014).
11. Chen, P., Kang, S., Li, C., Rupakheti, M., Yan, F., Li, Q. & Sillanpää, M. Characteristics and sources of polycyclic aromatic hydrocarbons in atmospheric aerosols in the Kathmandu Valley, Nepal. *Science of the Total Environment*. ***538***, 86-92 (2015).
12. Živković, M. M., Jovašević-Stojanović, M., Cvetković, A., Lazović, I., Tasic, V., Stevanović, Ž., & Gržetić, I. A. PAHs levels in gas and particle-bound phase in schools at different locations in Serbia. *Chemical Industry and Chemical Engineering Quarterly/CICEQ*. **21**(1), 159-167 (2015).
13. Hoseini, M., Yunesian, M., Nabizadeh, R., Yaghmaeian, K., Ahmadkhaniha, R., Rastkari, N. ... & Naddafi, K. Characterization and risk assessment of polycyclic aromatic hydrocarbons (PAHs) in urban atmospheric Particulate of Tehran, Iran. *Environmental Science and Pollution Research*. **23** (2), 1820-1832 (2016).
14. Teil, M. J., Moreau-Guigon, E., Blanchard, M., Alliot, F., Gasperi, J., Cladière, M. & Chevreuil, M. Endocrine disrupting compounds in gaseous and particulate outdoor air phases according to environmental factors. *Chemosphere*. **146**, 94-104 (2016).
15. Orakij, W., Chetiyanukornkul, T., Chuesaard, T., Kaganoi, Y., Uozaki, W., Homma, C. & Toriba, A. Personal inhalation exposure to polycyclic aromatic hydrocarbons and their nitro-derivatives in rural residents in northern Thailand. *Environmental monitoring and assessment*. **189** (10), 1-11 (2017).
16. Hamid, N., Syed, J. H., Junaid, M., Mahmood, A., Li, J., Zhang, G. & Malik, R. N. Elucidating the urban levels, sources and health risks of polycyclic aromatic hydrocarbons (PAHs) in Pakistan: Implications for changing energy demand. *Science of the Total Environment*. **619**, 165-175 (2018).
17. Chen, X. C., Chuang, H. C., Ward, T. J., Tian, L., Cao, J. J., Ho, S. S. H. & Ho, K. F. Indoor, outdoor, and personal exposure to PM2.5 and their bioreactivity among healthy residents of Hong Kong. *Environmental research*. **188**, 109780 (2020).
18. He, K., Xu, H., Feng, R., Shen, Z., Li, Y., Zhang, Y. & Cao, J. Characteristics of indoor and personal exposure to particulate organic compounds emitted from domestic solid fuel combustion in rural areas of northwest China. *Atmospheric Research*. **248**, 105181 (2021).
19. Jiang, Y., Hu, X., Yves, U. J., Zhan, H. & Wu, Y. Status, source and health risk assessment of polycyclic aromatic hydrocarbons in street dust of an industrial city, NW China. *Ecotoxicology and environmental safety*. **106**, 11-18 (2014).
20. Nguyen, T. C. *et al*. Polycyclic aromatic hydrocarbons in road-deposited sediments, water sediments, and soils in Sydney, Australia: comparisons of concentration distribution, sources and potential toxicity. *Ecotoxicology and Environmental Safety*. **104**, 339-348 (2014).
21. Kwon, H. O. & Choi, S. D. Polycyclic aromatic hydrocarbons (PAHs) in soils from a multi-industrial city, South Korea. *Science of the Total Environment*. **470**, 1494-1501 (2014).
22. Tue NM, Takahashi S, Suzuki G, Viet PH, Subramanian A, Bulbule KA, Tanabe S (2014) Methylated and unsubstituted polycyclic aromatic hydrocarbons in street dust from Vietnam and India: occurrence, distribution and in vitro toxicity evaluation. Environmental pollution 194:272-280Wang, J., Zhao, Z., Chen, J., Lu, H., Liu, G., Zhou, J. & Guan, X. PAHs accelerate the propagation of antibiotic resistance genes in coastal water microbial community. *Environmental Pollution*. **231**, 1145-1152 (2017).
23. Yu, B., Xie, X., Ma, L. Q., Kan, H. & Zhou, Q. Source, distribution, and health risk assessment of polycyclic aromatic hydrocarbons in urban street dust from Tianjin, China. *Environmental Science and Pollution Research*. **21** (4), 2817-2825 (2014).
24. Soltani, N., Keshavarzi, B., Moore, F., Tavakol, T., Lahijanzadeh, A. R., Jaafarzadeh, N. & Kermani, M. Ecological and human health hazards of heavy metals and polycyclic aromatic hydrocarbons (PAHs) in road dust of Isfahan metropolis, Iran. *Science of the Total Environment*. **505**, 712-723 (2015).
25. Wei, C., Bandowe, B. A. M., Han, Y., Cao, J., Zhan, C. & Wilcke, W. Polycyclic aromatic hydrocarbons (PAHs) and their derivatives (alkyl-PAHs, oxygenated-PAHs, nitrated-PAHs and azaarenes) in urban road dusts from Xi’an, Central China. *Chemosphere*. **134**, 512-520 (2015).
26. Ali, N., Ismail, I. M. I., Khoder, M., Shamy, M., Alghamdi, M., Costa, M. ... & Eqani, S. A. M. A. S. Polycyclic aromatic hydrocarbons (PAHs) in indoor dust samples from cities of Jeddah and Kuwait: levels, sources and non-dietary human exposure. *Science of the total environment*. **573**, 1607-1614 (2016).
27. Bandowe, B. A. M. & Nkansah, M. A. Occurrence, distribution and health risk from polycyclic aromatic compounds (PAHs, oxygenated-PAHs and azaarenes) in street dust from a major West African Metropolis. *Science of the Total Environment*. **553**, 439-449 (2016).
28. Keshavarzi, B., Abbasi, S., Moore, F., Mehravar, S., Sorooshian, A., Soltani, N. & Najmeddin, A. Contamination level, source identification and risk assessment of potentially toxic elements (PTEs) and polycyclic aromatic hydrocarbons (PAHs) in street dust of an important commercial center in Iran. *Environmental management*. **62** (4), 803-818 (2018).
29. Najmeddin, A., Moore, F., Keshavarzi, B. & Sadegh, Z. Pollution, source apportionment and health risk of potentially toxic elements (PTEs) and polycyclic aromatic hydrocarbons (PAHs) in urban street dust of Mashhad, the second largest city of Iran. *Journal of Geochemical Exploration*. **190**, 154-169 (2018).
30. Tong, R., Yang, X., Su, H., Pan, Y., Zhang, Q., Wang, J. & Long, M. Levels, sources and probabilistic health risks of polycyclic aromatic hydrocarbons in the agricultural soils from sites neighboring suburban industries in Shanghai. *Science of the Total Environment*. **616**, 1365-1373 (2018).
31. Liu, J., Zhang, J., Zhan, C., Liu, H., Zhang, L., Hu, T. & Qu, C. Polycyclic aromatic hydrocarbons (PAHs) in urban street dust of Huanggang, central China: Status, sources and human health risk assessment. *Aerosol and Air Quality Research*. **19** (2), 221-223 (2019).
32. Qishlaqi, A. & Beiramali, F. Potential sources and health risk assessment of polycyclic aromatic hydrocarbons in street dusts of Karaj urban area, northern Iran. *Journal of Environmental Health Science and Engineering*. **17** (2), 1029-1044 (2019).
